# The effectiveness and acceptability of physical activity interventions amongst older adults with lower socioeconomic status: a mixed methods systematic review

**DOI:** 10.1186/s12966-024-01666-8

**Published:** 2024-10-22

**Authors:** Danielle Harris, Schenelle Dayna Dlima, Ashley Gluchowski, Alex Hall, Emma Elliott, Luke Munford

**Affiliations:** 1 School of Health Sciences, Faculty of Biology, Medicine & Health, The University of Manchester, Manchester, M13 9PL UK; 2 School of Health & Society, University of Salford, Salford, M6 6PU UK; 3 National Institute for Health and Care Research, Applied Research Collaboration-Greater Manchester, School of Health Sciences, Faculty of Biology, Medicine and Health, The University of Manchester, Manchester, M13 9PL UK

**Keywords:** Physical activity, Older adults, Low socioeconomic status, Intervention development

## Abstract

**Background:**

Older adults with lower socioeconomic status are less likely to be physically active than those with higher socioeconomic status. To inform future intervention development, this review explored: [i] how effective are physical activity interventions at increasing levels of physical activity amongst older adults with lower socioeconomic status?; [ii] what factors are associated with the acceptability of physical activity interventions amongst older adults with lower socioeconomic status?; [iii] what are the implications for developing physical activity interventions for older adults with lower socioeconomic status?

**Methods:**

This mixed methods systematic review followed PRISMA guidelines. MEDLINE, CENTRAL, Embase, Scopus, Web of Science, PsycINFO, CINAHL, ASSIA and Sports Medicine and Education Index were searched up to May 2023, to identify quantitative, qualitative and mixed methods primary research studies measuring the effectiveness of and/or experiences of physical activity interventions for older adults (aged ≥ 65 years) with lower socioeconomic status. No limits on country were applied. Included studies were assessed for methodological quality using the Mixed Methods Appraisal Tool. Results were synthesised using a results-based convergent synthesis approach with narrative synthesis of quantitative findings and thematic synthesis of qualitative findings.

**Results:**

Thirty studies were included. Mixed effects were found for the effectiveness of physical activity interventions, with positive effects for increases in utilitarian walking (i.e. for transport) but not for leisure, mixed effects for objectively measured physical activity and no effects for self-reported total physical activity or muscle strengthening and flexibility activities. Engaging in physical activity interventions was perceived as offering many benefits, social familiarity was important to intervention acceptability and interventions were seen as more acceptable when they were compatible with the lifestyles of older adults with lower socioeconomic status.

**Conclusions:**

Future development of physical activity interventions for older adults with lower socioeconomic status should foster social connections, emphasise health benefits of physical activity, hold interventions in locations that are accessible and familiar to older adults with lower socioeconomic status, minimise costs to participants, employ individuals who share participant characteristics to lead interventions, and combine physical activity with other activities older adults with lower socioeconomic status already do to make more efficient use of time.

**Trial registration:**

PROSPERO CRD42023417312; https://www.crd.york.ac.uk/prospero/display_record.php?RecordID=417312.

**Supplementary Information:**

The online version contains supplementary material available at 10.1186/s12966-024-01666-8.

## Background

Physical activity is important for older adults; it provides numerous health benefits including improved cognitive function [1], frailty prevention [2], reduced risk of falls [3], reduced risk of cardiovascular disease [4, 5] as well as improvements to mental health [6], quality of life [7] and reduced mortality rates [8, 9]. The World Health Organization (WHO) recommends older adults (aged ≥ 65 years) perform at least 150–300 min of moderate-intensity aerobic activity or 75–100 min of vigorous-intensity aerobic activity per-week. They also recommend physical activity be multicomponent and include functional strength and balance training (on at least three days) to improve functional capacity and prevent falls [10]. Despite this, global figures show physical activity declines with increasing age [11], with 43.5% of older adults aged 60 years and over not meeting recommended levels of physical activity [12].

Socioeconomic status (SES) encompasses multiple factors including education, income, occupation, and area level deprivation [13, 14]. It is a key determinant of engaging in physical activity. Many studies show lower physical activity levels, particularly during leisure time, amongst those of lower SES [15–17]. These disparities persist into older age [17] and evidence shows similar patterns since the outbreak of COVID-19; older adults from lower SES groups had lower levels of activity during the pandemic [18–22]. Older adults from lower SES groups therefore represent a key target for public health interventions aimed at increasing physical activity.

Two key stages in developing public health interventions are identifying whether effective interventions exist for the target population and understanding the acceptability and priorities of that population [23]. Previous reviews have examined the effectiveness of physical activity interventions amongst older adults generally [24–26] and amongst lower SES samples of different ages [27, 28]. To the best of our knowledge, no reviews have examined the effectiveness of physical activity interventions at the intersection of both older and lower SES populations. Similarly, there is limited evidence regarding acceptability of physical activity interventions amongst lower SES older adults. A previous review [29] examined their acceptability amongst older adults more generally but did not look specifically at lower SES older adults. Some primary research studies have looked at acceptability of engaging in physical activity amongst older adults from lower SES groups [30–32]. These studies found factors such as deteriorating health, lack of belonging and loss of motivation, lack of available resources, negative social environment, and feeling vulnerable to violence and crime were barriers to engaging in more physical activity and less sedentary behaviours [30, 31]. Individuals also reported feeling undervalued and disadvantaged when comparing themselves to older adults living in higher SES areas, due to factors including lack of services and loss of local facilities, whilst physical activity was perceived as more acceptable when activities are enjoyable, familiar and address multiple needs such as social connection and leisure interests [32]. To the best of our knowledge, no reviews have evaluated and summarised findings examining the acceptability of physical activity interventions amongst older adults from lower SES groups.

### Aims

This mixed methods systematic review aimed to examine: [i] How effective are physical activity interventions at increasing levels of physical activity amongst older adults with lower SES [ii]? What factors are associated with the acceptability of physical activity interventions amongst older adults with lower SES [iii]? What are the implications for developing physical activity interventions for older adults with lower SES?

## Methods

### Protocol and registration

This review was conducted according to the Preferred Reporting Items for Systematic Reviews and Meta-Analyses (PRISMA) 2020 guidelines [33], the PRISMA checklist is available in Additional file 1. The protocol was registered on the International Prospective Register of Systematic Reviews (PROSPERO; CRD42023417312).

### Eligibility criteria

Eligibility criteria were developed using the Population, Intervention, Comparison, Outcomes and Study approach (PICOS; Table 1). Quantitative, qualitative and mixed methods primary research studies from any country which measured the effectiveness and/or experiences/acceptability of physical activity interventions for older adults from lower SES groups, with full-text versions published in English, were included.

**Table 1:** Study eligibility criteria

	Inclusion	Exclusion
Population	• Community-dwelling adults aged ≥ 65 years from low socioeconomic status (SES)^a^ groups/socioeconomically disadvantaged or deprived. • Studies with mixed SES samples were included provided that > 50% were low SES or results were stratified by SES and it was possible to extract low SES results separately.	• Average (mean or median) age of sample < 65years • Those not defined as being of low SES or socioeconomically disadvantaged or deprived. • Non-community dwelling (e.g. individuals living in nursing/care/residential homes or hospices, hospital inpatients, prison inmates)
Intervention	• Any intervention aimed at promoting, encouraging, increasing, or maintaining physical activity. • No limits on type of physical activity or how the intervention was delivered (i.e. self-directed or instructor led). • Interventions targeting multiple health behaviours (e.g. weight management) including physical activity where physical activity data could be separated.	• Interventions targeting multiple behaviours where effects of the physical activity component could not be separated.
Comparator	• Any comparator group who did not receive the physical activity intervention (e.g. usual care, wait-list control, health education)	• Studies which did not include a comparator group (as described in the column to the left).
Outcome	***Primary***: • Change in physical activity either self-reported (e.g. questionnaires, activity logs or diaries) or objectively measured (e.g. accelerometers, pedometers, smartwatches) • Measures of acceptability including participant experiences, barriers, and facilitators ***Secondary***: • Measures of physical function/fitness (e.g. gait speed, BMI, VO_2_ max, handgrip strength) • Psychological/wellbeing measures (e.g. fear of falling, anxiety, depression, quality of life)	• Studies which did not report at least one of the primary or secondary outcomes specified in the review protocol (listed in the column to the left).
Study type	• Quantitative, qualitative, and mixed methods primary interventional studies: - randomised controlled trials & quasi-experimental (assessing intervention effectiveness) - focus groups, interviews, surveys, questionnaires (assessing intervention experiences/acceptability)	• Systematic and non-systematic reviews • Individual case studies (*n* = 1) • Study protocols • Conference abstracts • Commentaries/editorials/ letters • Theses with no peer-reviewed publications

^a^A broad definition of lower socioeconomic status was used (including multiple factors such as education, income, occupation, and area deprivation). We accepted study authors’ definitions of low socioeconomic status/socioeconomic deprivation or disadvantage. If a study described a population group as lower SES, it was included

### Search strategy

Nine electronic databases were searched in May 2023: MEDLINE (Ovid), Cochrane Central Register of Controlled Trials (CENTRAL), Embase (Ovid), Scopus, Web of Science, PsycINFO (Ovid), Cumulative Index to Nursing and Allied Health Literature (CINAHL), Applied Social Sciences Index and Abstracts (ASSIA; ProQuest), and Sports Medicine and Education Index (ProQuest). No limits on date or country of publication were applied.

Search terms were based on previous reviews [24, 27, 29] and were discussed with a librarian. They included terms related to: [i] older adults [ii], lower SES [iii], physical activity [iv], study type, and [v] intervention effects and participant experience/acceptability. The search strategy is in Additional file 2.

Searches were supplemented with grey literature searches to identify interventions which may have been carried out by public or third sector bodies (e.g. local government or charity initiatives) or to identify potential further publications from theses. Five electronic sources were searched: Dissertations and Theses (ProQuest), Current Awareness Service for Health (CASH), Bielefeld Academic Search Engine (BASE), The King’s Fund Library, and Social Science Research Network (SSRN). Reference lists of all included studies were hand searched to identify any additional relevant studies.

### Data selection

After removing duplicates, titles and abstracts were screened independently by two reviewers using Rayyan software [34]. Full texts of potentially relevant papers were retrieved and screened independently by the same reviewers. Disagreements were resolved between the reviewers and in consultation with the review team.

### Data extraction

Data were extracted using a standardised form in Microsoft Excel by the primary reviewer and ten records were checked independently by the second reviewer: author; year of publication; study location; design; aim; participant characteristics (sample size, age, gender, ethnicity, socioeconomic status measures including education, income, occupation etc.); description of the intervention (type of physical activity, setting, mode of delivery, duration, intensity, any behaviour change frameworks/behaviour change techniques (BCTs) reported); details of comparator groups; measurements of primary and/or secondary outcomes.

### Quality assessment

Two reviewers independently assessed methodological quality of each included study using the Mixed Methods Appraisal Tool (MMAT) version 2018 [35]. Discrepancies were discussed between the reviewers and resolved in consultation with the review team. The MMAT discourages reporting an overall quality score for each study and recommends providing a presentation of ratings across the different quality domains (presented in Additional file 4 for each included study). While we did not exclude studies based on quality, we urge readers to interpret studies with low quality ratings with caution.

### Data synthesis

This review used a results-based convergent synthesis [36], where the quantitative and qualitative findings were first synthesised separately and then combined. For the first research question, quantitative findings were synthesised using narrative synthesis. Meta-analysis was not possible due to data heterogeneity. For the second research question, qualitative findings were synthesised using thematic synthesis [37]: [i] coding text [ii], developing descriptive themes, and [iii] generating analytical themes. Qualitative findings were extracted *ad verbatim* and imported into NVivo12. The first reviewer carried out inductive line-by-line coding of meaning and content. Potential descriptive themes and sub-themes were developed by grouping together similar codes. These were further developed into analytical themes by relating them back to the research question. Codes and themes were refined and discussed with the review team. Any quantitative data concerning acceptability and older adults’ experiences of physical activity interventions (i.e., from questionnaires or surveys) were synthesised via narrative synthesis. To answer the third research question, findings from the syntheses of effectiveness and acceptability were integrated narratively, focusing on what factors were acceptable to participants and whether these were effective at increasing physical activity to generate implications for developing future interventions.

## Results

### Study selection

**Fig. 1:**
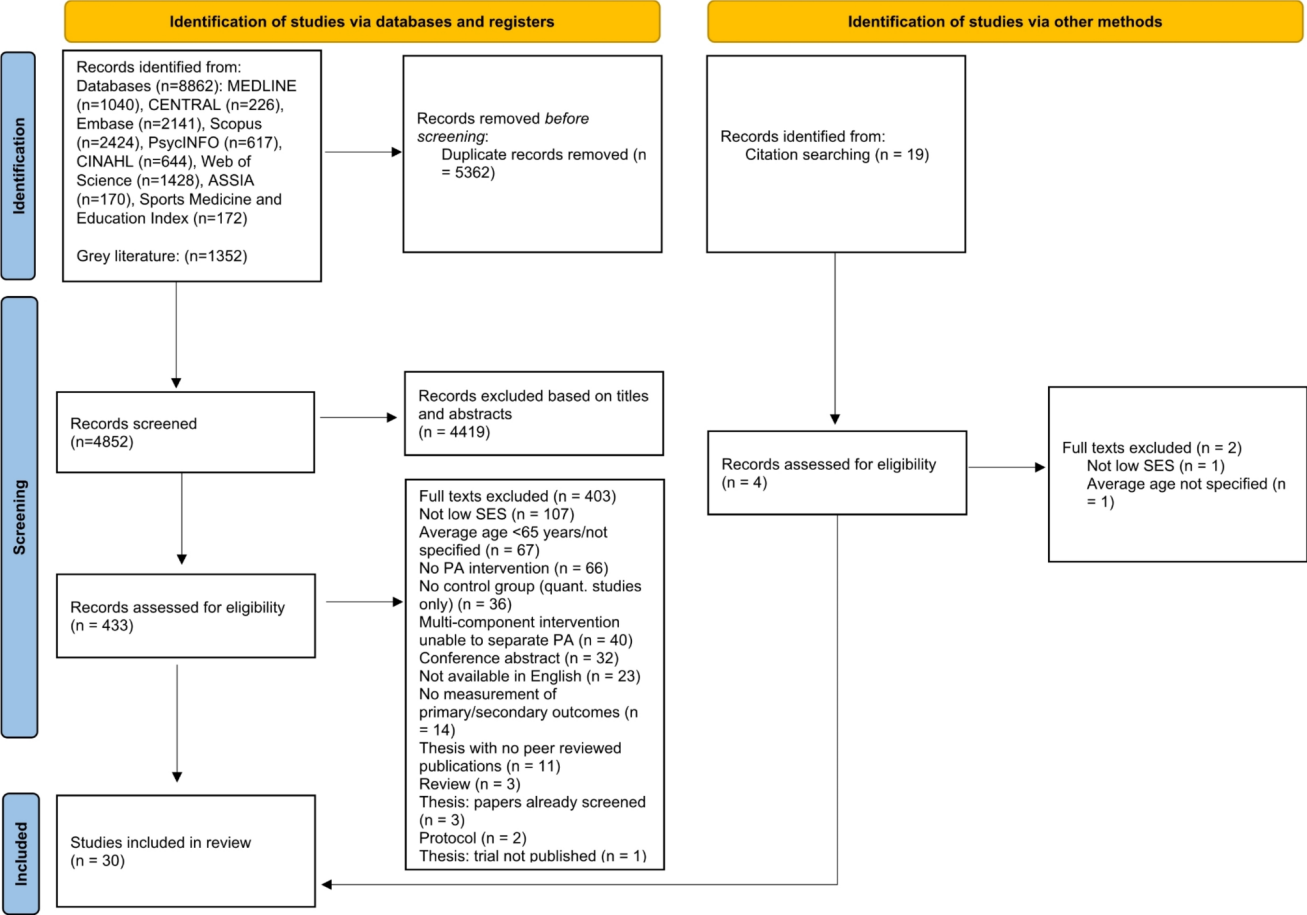
PRISMA Flow diagram of study selection

Figure 1 shows the study selection process: 4,852 unique records were retrieved, 433 full-texts were reviewed and 30 studies were included.

### Study characteristics

The 30 included studies comprised: 18 quantitative (14 RCTs [38–51] and four non-randomised studies [52–55]), six qualitative [56–61] and six mixed methods studies [62–67]. Most studies were conducted in the USA (20 studies [39, 40, 42, 44, 46–48, 50, 51, 54, 55, 57, 59–61, 63–67], three in the UK [49, 56, 62], two in Brazil [38, 41] and one each from Canada [58], The Netherlands [53], Poland [43], South Africa [45] and South Korea [52]. Studies were published from 1997 to 2023. Sample sizes ranged from *n* = 12 [60, 67] to *n* = 1635 [39]. See Additional file 3 for further study and participant characteristics.

### Quality assessment

Additional file 4 contains the full quality assessment for each study. All 30 studies included clear research questions and data to address these and so passed the initial screening stage of the MMAT. Five studies met all applicable MMAT criteria indicating higher study quality; one RCT [41] and four qualitative studies [56–59].

### Intervention characteristics

Full details of characteristics of the interventions in the included studies are in Additional file 5.

### Effectiveness of physical activity interventions amongst older adults with lower SES

#### Change in physical activity levels

Nine studies measured physical activity levels; six RCTs [40, 42, 44–46, 48, 49], two non-randomised studies [53, 54] and a mixed methods study in which the quantitative component used an RCT design [64]. Four other mixed methods studies also measured physical activity levels [62, 63, 66, 67]. However, as they did not include a comparator group who did not receive the intervention, they were not included in the quantitative data synthesis, only the qualitative synthesis. Mixed effects were found for the effectiveness of the interventions on physical activity levels. Full results for each study are available in Additional file 6.

#### Objectively measured physical activity

Three studies measured physical activity objectively using either Moderate-to-Vigorous Physical Activity (MVPA) minutes/day as measured by ActiGraph accelerometers [42, 64] or number of daily steps measured via Fitbit trackers [48]. There were contrasting effects for group walking interventions. One study found a significant increase in MVPA minutes/day over two years, for a twice-weekly peer health coach led intervention compared to a control group receiving health education, whose MVPA minutes/day decreased [42]. However, another study found no effect of a one-hour-per-week group walking intervention compared to a group advocacy skills programme [64]. No effect on daily steps was observed for a combined aerobic and strength and balance intervention [48].

#### Self-reported physical activity

Eight studies included self-reported measures of physical activity [40, 44–46, 48, 53, 54, 64].

##### Total physical activity

Most studies examining total self-reported physical activity found no effects. No effects on total physical activity were observed for an instructor-led group tai chi intervention [46], a combined aerobic and strength and balance group intervention [48], or interventions where participants chose their own activity plan [40, 54]. Two studies measured energy expenditure in kcal/week; one found no effect [54] whilst the other found a significant increase in exercise-related energy expenditure [45].

##### Walking

Positive effects were found for total walking and walking for a purpose (e.g. transport) but not walking for leisure. Significant increases in total weekly walking were found for both a designated walking route and a peer-led neighbourhood walking group compared to no intervention controls [53]. The same study also found an increase in utilitarian walking (i.e. walking for transport purposes), but no effect on walking for recreational purposes. Similar effects were found in another study using a group walking intervention, which increased walking for transportation but had no effect on leisure time walking [64]. Another study also observed similar effects with a significant increase in walking fast/briefly for exercise but no effect on walking leisurely for exercise or pleasure, after a virtual advisor intervention [44].

##### Strengthening and flexibility activities

One study measured levels of muscle strengthening and flexibility activities but found no effect on either measure at eight weeks [64].

### Secondary outcomes

Full results of secondary outcomes for each study are available in Additional file 7.

### Physical function

Sixteen studies measured physical function outcomes [38–43, 45–52, 55, 65].

#### Strength

Eight studies examined strength [38, 45, 47, 48, 50–52, 65]. Mixed effects were found for both upper and lower body strength. For lower limb strength, two studies employing strength and balance interventions found improvements on the Sit-to-Stand test (STS) [45, 50]. A third strength and balance intervention also found improvements but only for the fully supervised group (led by instructors) and not those who were minimally supervised (combination of instructor-led and at home exercises) [38]. No effect was found for yoga or dance interventions on STS scores [51, 52]. Contrasting effects were found for combined aerobic and strength and balance interventions on lower body strength as measured by 1-repetition maximum (1RM) on a leg press, with one study finding a significant increase [48] but another finding no effect [47].

For upper body strength, no significant effects were found for strength and balance interventions on grip strength [45, 50, 65]. No effect was found for a combined aerobic and strength and balance intervention on upper body strength measured by 1RM on a chest press [48]. However, a significant improvement in upper body strength was found for yoga [52].

#### Mobility

Six studies measured mobility [38, 39, 41, 43, 52, 65], this was mainly measured via the Timed Up and Go (TUG) and 400-metre walk test (400MWT). Significant improvements were found for combined aerobic and strength and balance interventions [41, 43], with no evidence that these benefits differed by education or income [39]. Significant improvements were also found for both a fully supervised and minimally supervised strength and balance intervention in TUG, but improvements for 400MWT were only found for the fully supervised group [38]. However, another strength and balance intervention found no effect on Performance Oriented Mobility Assessment short form scores [65]. There was also no effect of yoga or dance [52].

#### Balance

Balance was measured in five studies; significant improvements were found for dynamic balance [38, 43, 45] but no effects were found for static balance measures [43, 45, 51, 65].

#### Aerobic capacity/endurance

Four studies measured aerobic capacity/endurance; no significant effects were found for scores on the 6-Minute Walk Test (6MWT) [42, 45, 48]. However, one study did find significant improvements of a combined aerobic and strength and balance intervention on VO2 peak scores [47].

#### Gait speed

Three studies measured gait speed; there were no effects of a tai chi [46], combined aerobic and strength and balance intervention [48], or a strength and balance only intervention [45].

#### Flexibility

There were no significant effects on flexibility outcomes for yoga and dance interventions compared to controls [51, 52].

#### Multi-component physical function measures

Five studies examining multi-component measures found mixed effects. Two found no effects on Short Physical Performance Battery (SPPB) scores of a tai chi [46] and combined aerobic and strength and balance interventions [48], however one found a significant improvement in SPPB scores after a strength and balance only intervention [49]. Significant improvements were also found in all domains of Continuous Scale Physical Performance [47] and Senior Fitness Test scores [43] for combined aerobic and strength and balance interventions.

#### Self-reported physical functioning

Mixed effects were found for self-reported measures of physical function. Significant improvements in basic activities of daily living (ADLs) but not intermediate ADLs were found for a qigong intervention [55]. However, there were no effects on ADLs of a strength and balance intervention [45]. Two combined aerobic and strength training interventions found significant improvements in self-report physical functioning as measured by the Short Form Health Survey physical functioning scale (SF-36PF) [47, 48]. However, the latter found no effect on scores measuring risk of functional decline. Significant improvements in positive perceptions in change in physical functioning were found after a strength and balance intervention [65].

#### General physical health

Five studies which examined general physical health measures found no effect of physical activity interventions on HbA1c levels [40, 50], body composition (including muscle mass and waist to hip ratio) [50], general self-reported health status [45] or fall rates [46]. However, there were mixed effects for blood pressure and BMI with two studies finding no effect [42, 50], whilst one found a significant decrease in systolic but not diastolic blood pressure [45], the same study also found a significant decrease in BMI for one of the sites allocated to the strength and balance intervention but not the other site.

### Psychological/wellbeing outcomes

Eight studies measured psychological/wellbeing outcomes [41, 42, 44, 46, 48, 51, 54, 55].

#### Quality of life (QoL)

Six studies measured QoL [41, 42, 46, 48, 54, 55]. Significant improvements in different aspects of QoL were found for combined aerobic and strength and balance [41, 48] and group walking [42]. Mixed effects were found for qigong/tai chi; one study found an improvement on physical component QoL scores but not mental component [55], whilst another found no effect on either [46]. There were mixed effects on different domains of health-related QoL (HRQoL) after an intervention where individuals chose their own physical activity plan; with significant improvement in self-esteem scores found but no effects on the other domains of physical functioning, limitations in social activities due to health, energy, pain, sleep adequacy, self-rated health, sense of mastery, psychological wellbeing, and life satisfaction [54].

#### Depression

There were no significant effects of any interventions on depression [42, 46]. One study found an improvement after a yoga intervention but there were no significant differences compared to the socialisation control group [51].

#### Other psychological/wellbeing outcomes

One study found a significant improvement in motivational measures after an artificial intelligence (AI) virtual advisor walking intervention based on Social Cognitive Theory and the Transtheoretical Model including: understanding risks of inactivity, committing oneself to being physically active, substituting more active alternatives, rewarding oneself for being physically active and reminding oneself to be physically active. No effects were found for understanding the benefits of a physically active lifestyle or enlisting social support to be physically active [44].

There were no effects found for other outcomes including loneliness [51], sleep quality [48], measures of balance confidence [46], morale [51], hope [51] and chronic pain [55].

### Acceptability of physical activity interventions amongst older adults with lower SES

#### Quantitative findings

Two studies [44, 55] examined quantitative measures of intervention acceptability. Both found high levels of acceptability; in terms of overall participant satisfaction with a qigong intervention [55] and in participant perceptions of trustworthiness of information being delivered and satisfaction with the relationship between virtual advisor and participant, in a digitally delivered walking intervention [44].

#### Qualitative findings

**Fig. 2:**
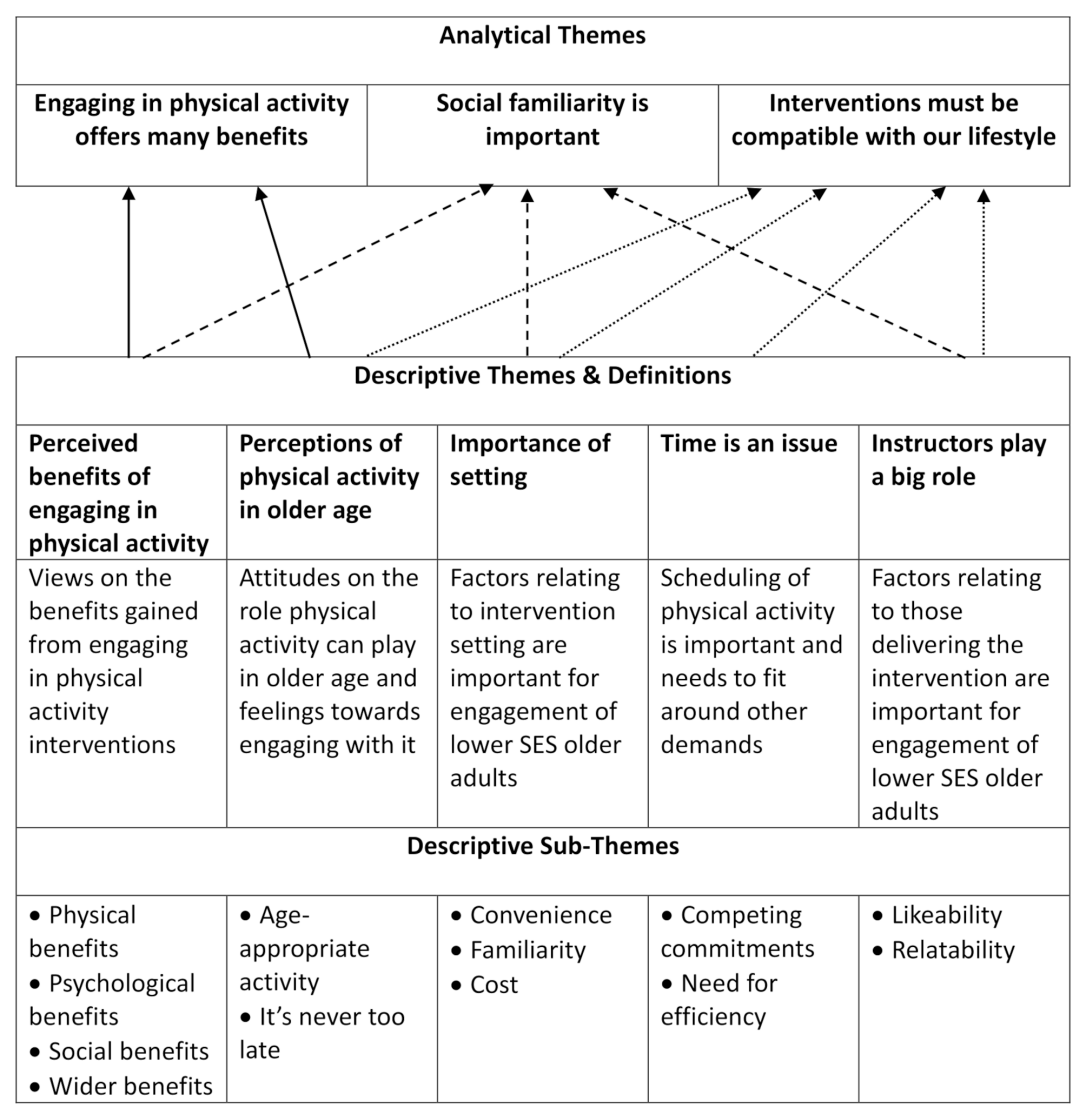
An overview of analytical themes and the descriptive themes from which they were derived

Eleven studies [56–63, 65–67] were included in the thematic synthesis. An overview of the themes identified is provided (Fig. 2) alongside a more detailed description of the descriptive themes with supporting findings and quotations (Additional file 8). Five descriptive themes were identified: Perceived benefits of engaging in physical activity; Perceptions of physical activity in older age; Importance of setting; Time is an issue; Instructors play a big role. These themes were developed into three analytical themes: Engaging in physical activity offers many benefits; Social familiarity is important; Interventions must be compatible with our lifestyle, which illustrate factors affecting acceptability of physical activity interventions amongst older adults from lower socioeconomic groups.

#### Theme 1: Engaging in physical activity offers many benefits

Participants talked about many different benefits from engaging in physical activity interventions, including physical health benefits such as improvements in strength, mobility, flexibility and balance, weight loss, increased energy, and helping with existing health issues. Psychological benefits were also described including improvements in mood, confidence, relaxation, and sleep. There were also social benefits such as meeting new people and making new friends, connections which often extended beyond the intervention. Wider benefits of engaging in physical activity were reported, for example engaging in walking as active transport and increased knowledge of opportunities to be active within their local area. Participants perceived the importance of physical activity in older age and expressed that it is never too late to obtain the health benefits of doing more activity and that this was something they had the power to do.

#### Theme 2: Social familiarity is important

Social familiarity was a predominant factor contributing to intervention acceptability. This extended beyond making social connections as described in theme 1, to include being amongst peers with similar characteristics which gave a social relatability to the intervention setting and was a facilitator to participants engaging in the interventions. Having an instructor participants could connect to and relate to through shared understanding and experiences was also seen as an important factor related to acceptability. Peer leaders were seen as an effective solution to this.

#### Theme 3: Interventions must be compatible with our lifestyle

Participants described through various factors their needs and preferences for engaging in physical activity interventions. Many described the difficulties of scheduling physical activity alongside other commitments and demands on their time including caring for others and attending medical appointments. They expressed that combining physical activity alongside other activities was a solution to overcoming these barriers and helping to make more efficient use of their time.

Participants also described the importance of physical activity classes being convenient. Having a location that was easy to get to was a facilitator to intervention engagement, whilst longer travel distances was a barrier to participation. The familiarity of the physical setting itself was also important to participants. Cost was also a barrier to participation, both in terms of the cost of physical activity sessions themselves or the cost of transport to reach them. Providing physical activity interventions free of charge was a facilitator to engagement. Other preferences were also reported regarding the type of physical activity, some participants reported gentle exercise as being more age appropriate and this was observed as a draw for engaging with the intervention initially, rather than more traditional gym-based exercise.

### Implications for developing physical activity interventions for older adults with lower SES

Based on the integration of findings from the data syntheses examining the effectiveness and acceptability of physical activity interventions amongst older adults from lower SES groups, we propose six implications for consideration when developing future physical activity interventions for this population. Further details are found in Additional file 9.

1. ***Interventions should consider how to foster social connectivity***: Physical activity interventions could provide opportunities for participants to mix with others, particularly with those they share experiences with.
2. ***Interventions should consider how to emphasise the benefits of physical activity***: Future interventions could emphasise the different benefits that can be gained from engaging in physical activity to improve both intervention acceptability and effectiveness.
3. ***Physical activity could be combined with other activities to make more efficient use of participants’ time***: Future development of physical activity interventions could carefully plan the scheduling of physical activity programmes. For example, combining physical activity with other activities lower SES older adults already do or targeting physical activity for other purposes (e.g. transportation).
4. ***Interventions should consider using locations that are accessible and familiar***: Future interventions could be held in settings that are easy to access to reduce barriers of longer travel distances, and settings lower SES older adults are already accustomed to rather than less familiar settings like gyms.
5. ***Interventions should consider how to minimise costs to participants***: Future interventions could minimise costs to those taking part, for example through providing sessions free of charge and reducing travel costs.
6. ***Interventions should consider using leaders who have shared characteristics with participants***: Future interventions could be delivered by peer leaders.

## Discussion

The aim of this review was to examine the effectiveness and acceptability of physical activity interventions amongst older adults with lower SES, as well as developing implications for future development of physical activity interventions amongst this population.

This review found mixed effectiveness of physical activity interventions on change in physical activity levels. Positive effects were found for increases in walking; however, this was found for walking for a purpose (i.e. transportation) and not walking for leisure. There were mixed effects for objectively measured physical activity, with some studies showing positive effects whilst others reported no effects. No effects were found for self-reported total physical activity or for time spent doing muscle strengthening and flexibility activities.

Regarding acceptability, participants described how engaging in physical activity interventions offered many benefits and how it was never too late to obtain these. Reported benefits were physical, psychological, and social as well as other wider benefits like increased physical activity through walking as transportation and increased knowledge of local opportunities for physical activity. Previous qualitative research also found that recognising the health benefits of exercise was the main reason for joining a multicomponent health promotion programme for lower income older adults [68].

Social connection was a major factor contributing to intervention acceptability and this was often a motivator for individuals engaging with the intervention initially. This is line with a previous review looking at acceptability of physical activity amongst older adults [29]. Another review looking at effectiveness of physical activity interventions amongst lower SES groups also found positive effects for group-based physical activity [28]. Other research looking at engagement in physical activity amongst lower SES older adults also found similar importance of social factors [22, 30, 32].

Being amongst peers with whom they shared similar experiences (both other participants and intervention leaders) was a facilitator to engagement in physical activity interventions. This contrasts with findings from another systematic review looking at effectiveness of physical activity interventions amongst older adults which found no moderation effect of the type of individual delivering the intervention [26]. This suggests that lower SES older adults may have different needs to the wider population of older adults when it comes to engaging in physical activity interventions.

Another key factor associated with intervention acceptability was that interventions had to be compatible with the lifestyles of lower SES older adults; locations of physical activity programmes which were convenient and familiar were facilitators. In contrast, there was no moderating effect of delivery setting on the effectiveness of physical activity interventions amongst the wider population of older adults [26], again suggesting that lower SES older adults have different needs compared to other older adults.

Competing commitments and time demands were often reported as barriers to engagement, with participants wanting to combine physical activity with other activities to make more efficient use of their time. This is in line with other qualitative research which found that having other priorities was a major barrier to engaging in a multicomponent health promotion programme for low-income older adults [68]. Physical activity was also perceived as more acceptable when activities are enjoyable, familiar and addressed multiple needs such as social connection and leisure interests [32].

Based on the findings regarding effectiveness and acceptability of physical activity interventions amongst lower SES older adults, six implications were proposed for the future development of physical activity interventions for this group: interventions should consider how to foster social connectivity; emphasise the benefits of physical activity; use locations that are accessible and familiar to lower SES older adults, minimise costs to participants, intervention leaders could have shared characteristics with participants (i.e. peer leaders), and physical activity could be combined with other activities to make more efficient use of participants’ time.

### Strengths and limitations

To our knowledge, this is the first systematic review evaluating the effectiveness and acceptability of physical activity interventions amongst older adults from lower SES groups. A strength of this review is its rigorous systematic methods and comprehensive search strategy. Mixed methods reviews allow for the synthesis of evidence regarding complex interventions [69] and try to maximise findings of different forms of research evidence to help inform policy and practice [70]. The mixed methods design of this review allowed for the integration of both quantitative and qualitative findings, to better understand the mechanisms behind intervention effects more efficiently than having individual reviews looking at effectiveness and acceptability separately.

However, in terms of limitations, we were unable to conduct a meta-analysis due to the heterogeneity of study designs and were not able to gain an overall effect of the effectiveness of physical activity interventions amongst lower SES older adults. Only five of the included studies met all quality criteria of the MMAT indicating higher study quality, of which only one of these was an RCT. In line with MMAT guidance, studies were not excluded based on quality; however, caution should be applied when interpreting findings from this review as studies scoring lower on MMAT quality criteria were given the same weight as findings scoring more highly. Another limitation of this review is that it did not analyse the ‘active’ components of the interventions by not formally coding BCTs [71]. Often BCTs were poorly reported within the studies. This review also did not analyse other factors contributing to intervention effectiveness such as dose and session frequency.

### Implications for future research

There is evidence to suggest that lower SES older adults may have different needs/preferences to those of older adults more generally when it comes to engaging in physical activity interventions. More research is needed to further explore differences in effectiveness and acceptability of physical activity interventions between lower SES older adults and the wider population of older adults. Higher quality RCTs are also needed to examine the effectiveness of physical activity interventions amongst older adults with lower SES. Future research should also seek to analyse active components to better understand the effectiveness of physical activity interventions amongst lower SES older adults using the Behaviour Change Technique Taxonomy (BCTT; [72]) to code BCTs, and should also seek to investigate the effects of other factors such as dose and session frequency.

Although ethnicity was not the focus of this review, we found little difference with regards to acceptability of physical activity interventions between those studies with higher ethnic diversity and majority ethnic minority samples compared to those with majority White samples. Only one study [63] reported that participants felt talking to someone of a different race to them was a barrier to participating in the intervention. The lack of ethnic differences in this review is in contrast with previous research reporting language and cultural barriers to participation in physical activity amongst older adults of different ethnicities [73, 74]. Future research should further explore any differences in the acceptability of engaging in physical activity amongst lower SES older adults of different ethnicities, to inform the development of interventions that are culturally competent. It is also important to note that the included studies had predominantly female samples, future research should seek to include more males and explore whether there are gender differences with regards to the effectiveness and acceptability of physical activity interventions amongst older adults of lower SES.

Most of the included studies were conducted prior to the pandemic, with research showing that lower SES older adults were less likely to be physically active compared to higher SES older adults during the COVID-19 pandemic [18–22], future research should also look to investigate whether the pandemic has had any impact on the acceptability of engaging in physical activity amongst lower SES older adults. Future research should also consider involving older adults from lower SES groups in the development of physical activity interventions.

## Conclusions

Mixed effects were found regarding the effectiveness of physical activity interventions amongst lower SES older adults. Positive effects were found for increases in utilitarian walking (i.e. for transport) but not for leisure. Whilst there were mixed effects for objectively measured physical activity and no effects for self-reported total physical activity or time spent doing muscle strengthening and flexibility activities.

Lower SES older adults perceived engaging in physical activity interventions as having many benefits. Social familiarity was important to intervention acceptability and was often a motivator for individuals engaging with the intervention initially. Physical activity interventions were seen as more acceptable when they were compatible with the lifestyles of lower SES older adults. Participants valued convenient locations that were familiar to them and felt that combining physical activity with other activities could help to overcome barriers of competing schedule commitments.

Older adults from lower SES groups are at greater risk of physical inactivity and as such are important targets for interventions aimed at increasing physical activity. Future development of physical activity interventions for this group should consider fostering social connections, emphasising the health benefits of physical activity, holding interventions in locations that are accessible and familiar to lower SES older adults, minimising costs to participants, employing individuals who share participant characteristics to lead interventions and combining physical activity with other activities lower SES older adults already do to help make more efficient use of their time.

## Electronic supplementary material

Below is the link to the electronic supplementary material.

Additional file 1: PRISMA 2020 Checklist

Additional file 2: Search strategy (12/05/2023)

Additional file 3: Characteristics of included studies and populations

Additional file 4: Quality assessment of studies using Mixed Methods Appraisal Tool (MMAT), version 2018 criteria

Additional file 5: Characteristics of included interventions, study outcomes and which review question each addressed

Additional file 6: Quantitative data from studies included in systematic review synthesis

Additional file 7: Quantitative data for secondary outcomes from studies included in systematic review synthesis

Additional file 8: Descriptive themes, sub-themes and supporting findings and quotes identified in the thematic synthesis

Additional file 9: Implications for intervention development and findings from which they were derived

## Data Availability

All data generated or analysed during this study are included in this published article [and its supplementary information files].

## Abbreviations

1RM: 1-repetition maximum
400MWT: 400-metre walk test
6MWT: 6-minute walk test
ADLs: Activities of daily living
ASSIA: Applied Social Sciences Index
BASE: Bielefeld Academic Search Engine
BCTs: Behaviour change techniques
CASH: Current Awareness Service for Health
CENTRAL: Cochrane Central Register of Controlled Trials
CINAHL: Cumulative Index to Nursing and Allied Health Literature
HRQoL: Health-related quality of life
MMAT: Mixed Methods Appraisal Tool
MVPA: Moderate-to-Vigorous Physical Activity
PICOS: Population, Intervention, Comparison, Outcomes, Study
PRISMA: Preferred Reporting Items for Systematic Reviews and Meta-Analyses
QoL: Quality of life
RCT: Randomised controlled trial
SES: Socioeconomic status
SPPB: Short Physical Performance Battery
SSRN: Social Science Research Network
STS: Sit-to-Stand test
WHO: World Health Organization
