# Supplementary material for: The effectiveness and acceptability of physical activity interventions amongst older adults with lower socioeconomic status: a mixed methods systematic review
Source: Int J Behav Nutr Phys Act. 2024 Oct 22;21:121. doi: 10.1186/s12966-024-01666-8 (PMC11495005; doi:10.1186/s12966-024-01666-8)
Supplement: Supplementary file 2 — Additional file 2: Search strategy (12/05/2023) [file 12966_2024_1666_MOESM2_ESM.docx]

Additional file 2: Search strategy (12/05/2023)

MEDLINE (R) ALL

Embase

PsycINFO

1. older adult*.mp.

2. older people.mp.

3. elder*.mp.

4. senior*.mp.

5. later life.mp.

6. ag$ing.mp.

7. geriatric.mp.

8. 1 or 2 or 3 or 4 or 5 or 6 or 7

9. depriv*.mp.

10. poverty.mp.

11. low* income.mp.

12. disadvantaged.mp.

13. low SES.mp.

14. low* socioeconomic.mp.

15. low* educat*.mp.

16. social class.mp.

17. social status.mp.

18. 9 or 10 or 11 or 12 or 13 or 14 or 15 or 16 or 17

19. physical activity.mp.

20. exercise.mp.

21. sport.mp.

22. resistance training.mp.

23. aerobic activ*.mp.

24. strength training.mp.

25. walk*.mp.

26. yoga.mp.

27. danc*.mp.

28. cycling.mp.

29. swim*.mp.

30. tai chi.mp.

31. gym.mp.

32. activities of daily living.mp.

33. balance training.mp.

34. cardiovascular activ*.mp.

35. endurance activ*.mp.

36. functional training.mp.

37. lifestyle activ*.mp.

38. qigong.mp.

39. chi kung.mp.

40. recreational activ*.mp.

41. 19 or 20 or 21 or 22 or 23 or 24 or 25 or 26 or 27 or 28 or 29 or 30 or 31 or 32 or 33 or 34 or 35 or 36 or 37 or 38 or 39 or 40

42. intervention*.mp.

43. RCT.mp.

44. randomised control* trial.mp.

45. trial.mp.

46. program*.mp.

47. evaluation*.mp.

48. implementation.mp.

49. experiment*.mp.

50. qualitative.mp.

51. mixed method*.mp.

52. interview*.mp.

53. focus group*.mp.

54. survey*.mp.

55. questionnaire.mp.

56. 42 or 43 or 44 or 45 or 46 or 47 or 48 or 49 or 50 or 51 or 52 or 53 or 54 or 55

57. effect*.mp.

58. affect*.mp.

59. efficacy.mp.

60. impact*.mp.

61. acceptab*.mp.

62. feasibility.mp.

63. perception*.mp.

64. belief*.mp.

65. experience.mp.

66. attitude.mp.

67. barrier*.mp.

68. motivat*.mp.

69. 57 or 58 or 59 or 60 or 61 or 62 or 63 or 64 or 65 or 66 or 67 or 68

70. 8 and 18 and 41 and 56 and 69

CENTRAL

Title-Abstract-Keyword

#1 "older adult*" OR "older people" OR elder* OR senior* OR "later life" OR ag?ing OR geriatric

#2 depriv* OR poverty OR "low* income" OR disadvantaged OR "low SES" OR "low* socioeconomic" OR "low* educat*" OR "social class" OR "social status"

#3 "physical activity" OR exercise OR sport OR "resistance training" OR "aerobic activ*" OR "strength training" OR walk* OR yoga OR danc* OR cycling OR swim* OR "tai chi" OR gym OR "activities of daily living" OR "balance training" OR "cardiovascular activ*" OR "endurance activ*" OR "functional training" OR "lifestyle activ*" OR qigong OR "chi kung" OR "recreational activ*"

#4 intervention* OR RCT OR "randomised control* trial" OR trial OR program* OR evaluation OR implementation OR experiment* OR qualitative OR "mixed method*" OR interview* OR "focus group*" OR survey* OR questionnaire

#5 effect* OR affect* OR efficacy OR impact* OR acceptab* OR feasibility OR perception* OR belief* OR experience OR attitude OR barrier* OR motivat*

#6 #1 AND #2 AND #3 AND #4 AND #5

Scopus

TITLE-ABS-KEY ( ( "older adult*" OR "older people" OR elder* OR senior* OR "later life" OR ag?ing OR geriatric ) AND ( depriv* OR poverty OR "low* income" OR disadvantaged OR "low SES" OR "low* socioeconomic" OR "low* educat*" OR "social class" OR "social status" ) AND ( "physical activity" OR exercise OR sport OR "resistance training" OR "aerobic activ*" OR "strength training" OR walk* OR yoga OR danc* OR cycling OR swim* OR "tai chi" OR gym OR "activities of daily living" OR "balance training" OR "cardiovascular activ*" OR "endurance activ*" OR "functional training" OR "lifestyle activ*" OR qigong OR "chi kung" OR "recreational activ*" ) AND ( intervention* OR rct OR "randomised control* trial" OR trial OR program* OR evaluation OR implementation OR experiment* OR qualitative OR "mixed method*" OR interview* OR "focus group*" OR survey* OR questionnaire ) AND ( effect* OR affect* OR efficacy OR impact* OR acceptab* OR feasibility OR perception* OR belief* OR experience OR attitude OR barrier* OR motivat* ) )

Web of Science

"older adult*" OR "older people" OR elder* OR senior* OR "later life" OR ag*ing OR geriatric (Topic) and depriv* OR poverty OR "low* income" OR disadvantaged OR "low SES" OR "low* socioeconomic" OR "low* educat*" OR "social class" OR "social status" (Topic) and "physical activity" OR exercise OR sport OR "resistance training" OR "aerobic activ*" OR "strength training" OR walk* OR yoga OR danc* OR cycling OR swim* OR "tai chi" OR gym OR "activities of daily living" OR "balance training" OR "cardiovascular activ*" OR "endurance activ*" OR "functional training" OR "lifestyle activ*" OR qigong OR "chi kung" OR "recreational activ*" (Topic) and intervention* OR RCT OR "randomised control* trial" OR trial OR program* OR evaluation OR implementation OR experiment* OR qualitative OR "mixed method*" OR interview* OR "focus group*" OR survey* OR questionnaire (Topic) and effect* OR affect* OR efficacy OR impact* OR acceptab* OR feasibility OR perception* OR belief* OR experience OR attitude OR barrier* OR motivat* (Topic)

CINAHL
( "older adult*" OR "older people" OR elder* OR senior* OR "later life" OR ag$ing OR geriatric ) AND ( depriv* OR poverty OR "low* income" OR disadvantaged OR "low SES" OR "low* socioeconomic" OR "low* educat*" OR "social class" OR "social status" ) AND ( "physical activity" OR exercise OR sport OR "resistance training" OR "aerobic activ*" OR "strength training" OR walk* OR yoga OR danc* OR cycling OR swim* OR "tai chi" OR gym OR "activities of daily living" OR "balance training" OR "cardiovascular activ*" OR "endurance activ*" OR "functional training" OR "lifestyle activ*" OR qigong OR "chi kung" OR "recreational activ*" ) AND ( intervention* OR RCT OR "randomised control* trial" OR trial OR program* OR evaluation OR implementation OR experiment* OR qualitative OR "mixed method*" OR interview* OR "focus group*" OR survey* OR questionnaire ) AND ( effect* OR affect* OR efficacy OR impact* OR acceptab* OR feasibility OR perception* OR belief* OR experience OR attitude OR barrier* OR motivat* )

ASSIA

Sports Medicine and Education Index

(title("older adult*" OR "older people" OR elder* OR senior* OR "later life" OR ag?ing OR geriatric) OR abstract("older adult*" OR "older people" OR elder* OR senior* OR "later life" OR ag?ing OR geriatric) OR subject("older adult*" OR "older people" OR elder* OR senior* OR "later life" OR ag?ing OR geriatric)) AND (title(depriv* OR poverty OR "low* income" OR disadvantaged OR "low SES" OR "low* socioeconomic" OR "low* educat*" OR "social class" OR "social status") OR abstract(depriv* OR poverty OR "low* income" OR disadvantaged OR "low SES" OR "low* socioeconomic" OR "low* educat*" OR "social class" OR "social status") OR subject(depriv* OR poverty OR "low* income" OR disadvantaged OR "low SES" OR "low* socioeconomic" OR "low* educat*" OR "social class" OR "social status")) AND (title("physical activity" OR exercise OR sport OR "resistance training" OR "aerobic activ*" OR "strength training" OR walk* OR yoga OR danc* OR cycling OR swim* OR "tai chi" OR gym OR "activities of daily living" OR "balance training" OR "cardiovascular activ*" OR "endurance activ*" OR "functional training" OR "lifestyle activ*" OR qigong OR "chi kung" OR "recreational activ*") OR abstract("physical activity" OR exercise OR sport OR "resistance training" OR "aerobic activ*" OR "strength training" OR walk* OR yoga OR danc* OR cycling OR swim* OR "tai chi" OR gym OR "activities of daily living" OR "balance training" OR "cardiovascular activ*" OR "endurance activ*" OR "functional training" OR "lifestyle activ*" OR qigong OR "chi kung" OR "recreational activ*") OR subject("physical activity" OR exercise OR sport OR "resistance training" OR "aerobic activ*" OR "strength training" OR walk* OR yoga OR danc* OR cycling OR swim* OR "tai chi" OR gym OR "activities of daily living" OR "balance training" OR "cardiovascular activ*" OR "endurance activ*" OR "functional training" OR "lifestyle activ*" OR qigong OR "chi kung" OR "recreational activ*")) AND (title(intervention* OR RCT OR "randomised control* trial" OR trial OR program* OR evaluation OR implementation OR experiment* OR qualitative OR "mixed method*" OR interview* OR "focus group*" OR survey* OR questionnaire) OR abstract(intervention* OR RCT OR "randomised control* trial" OR trial OR program* OR evaluation OR implementation OR experiment* OR qualitative OR "mixed method*" OR interview* OR "focus group*" OR survey* OR questionnaire) OR subject(intervention* OR RCT OR "randomised control* trial" OR trial OR program* OR evaluation OR implementation OR experiment* OR qualitative OR "mixed method*" OR interview* OR "focus group*" OR survey* OR questionnaire)) AND (title(effect* OR affect* OR efficacy OR impact* OR acceptab* OR feasibility OR perception* OR belief* OR experience OR attitude OR barrier* OR motivat*) OR abstract(effect* OR affect* OR efficacy OR impact* OR acceptab* OR feasibility OR perception* OR belief* OR experience OR attitude OR barrier* OR motivat*) OR subject(effect* OR affect* OR efficacy OR impact* OR acceptab* OR feasibility OR perception* OR belief* OR experience OR attitude OR barrier* OR motivat*))
