# Supplementary material for: The effectiveness and acceptability of physical activity interventions amongst older adults with lower socioeconomic status: a mixed methods systematic review
Source: Int J Behav Nutr Phys Act. 2024 Oct 22;21:121. doi: 10.1186/s12966-024-01666-8 (PMC11495005; doi:10.1186/s12966-024-01666-8)
Supplement: Supplementary file 3 — Additional file 3: Characteristics of included studies and populations [file 12966_2024_1666_MOESM3_ESM.docx]

**Additional file 3. Characteristics of included studies and populations.**

| **Study** | **Location** | **Design** | **Sample size, N** | **Age (years),**  **Mean (SD)** | **Gender, % female** | **SES** | **Ethnicity** |
| --- | --- | --- | --- | --- | --- | --- | --- |
| Almeida et al 2013, | Sao Paulo, Brazil | RCT | 76 | 79.06 (4.55) | 82.9% | - Monthly income Brazilian Reais, mean (SD)^a^   Fully supervised = 487 (367)  Minimally supervised = 415 (423)  Con = 457 (638)   - Years of formal education, mean (SD):   Fully supervised = 3.9 (2.2)  Minimally supervised = 3.8 (2.55)  Con = 4.0 (3.8) | Not reported |
| Bann et al., 2016^b^ | USA | RCT | 1635 | 78.9 (5.2) | 67.2% | Education:  0.8% No formal  2.0% Elementary school  29.6% High school/equivalent  39.2% College  24.6% Postgraduate  3.5% Other  0.3% Unknown | 75.8% White  17.6% African American/Black  3.7% Latino/Hispanic/Spanish  0.9% Asian  1.6% other/mixed  0.3% refused/missing |
| Batik et al, 2008 | Seattle,  USA | RCT | 305 | Immediate Intervention group = 73.6 (7.9)  Delayed Intervention group = 71.9 (6.3) | Immediate Intervention group: 71.9%  Delayed Intervention group: 65.9% | Recruited from neighbourhood below median income for Seattle and King County | Immediate Intervention group: 80.6% Non-white  Delayed Intervention group: 85.9% Non-white |
| Brandao et al, 2021 | Senhor do Bonfim, Brazil | RCT | 125 | 68 (7) | 88% | - Low income 78% ≤ 2 SM - Education: 76% ≤ 3yrs of study | Not reported |
| Britten et al, 2023 | Yorkshire, UK | Mixed Methods;  Uncontrolled design,  Focus groups | 685 | 75.8 (9.4) | 86% | IMD quintiles:  37.8% 1st (most deprived)  16.1% 2nd  16.4% 3rd  17.7% 4th  7.7% 5th (least deprived) | 81.9% White British |
| Crist et al, 2022 | San Diego County, USA | RCT | 476 | 71.0 (8.9) | 75.7% | - 59.3% Low income (Household income below 80% of the Area Median Income) - 47.0% college education or above | 62.4% White  24.6% Black  4.4% Asian  8.6% Other |
| Cwirlej-Sozanska et al, 2018 | Rzeszow, Poland | RCT | 50 | Int = 67.62 (3.68)  Con = 67.50 (3.64) | Int: 71.43%  Con: 91.30% | - Income: <261 euros per month - Education:   Primary  Int: 14.29%; Con: 13.04%  Secondary  Int: 42.86%; Con: 56.53%  Higher  Int: 47.62%; Con: 30.43% | Not reported |
| Evans et al, 2018 | Scotland, UK | Qualitative;  Interviews | 18 | Median = 65 | 100% | - 54% lived in areas in two most deprived quintiles (according to Scottish IMD) - n=7 had no formal qualifications | Not reported |
| Hammerback et al, 2012 | Seattle, USA | Mixed Methods;  Pre-post design,  Semi-structured interviews | 131 | 70 | Not reported | - Recruited from low-income neighbourhood in Seattle - Education: 29% college graduate | 51% white |
| King et al, 2013 | San Jose, USA | RCT | 40 | 68.3 (8.2) | 72.5% | - Education, year:   33.3% ≤8  12.8% 9-11  25.7% High school  28.2% Some college+   - Household income: 81% < $50,000 (classed as low income, less than 80% of AMI)   30.0% <$15,000  17.5% $15,000-24,999  22.5% $25,000-$34,999  2.5% $35,000-$49,999  12.5% $50,000-74,999  5.0% ≥$75,000  10% Refused   - Occupation:   30.0% Professional/manager  22.5% Clerical  10.0% Service  12.5% Skilled craft  25.0% Laborers | 92.5% Latino |
| Kolbe-Alexander et al, 2006 | South Africa | RCT | 81 | 68 | 100% | - From socioeconomically disadvantaged communities - Most receiving state pension | Mixed racial ancestry - not further specified |
| Lee et al, 2016 | Yeongdo-gu, Busan City, South Korea | Non-randomised | 60 | Yoga =71.62(5.51)  Dance =72.06(4.55)  Sports massage =74.36(4.06) | 100% | - Household income <120% of average monthly household income - Also subject to protection system (basic old-age pension) or belonged to household entitled to basic support from local government | Not reported |
| Lipsitz et al, 2019 | Boston, USA | Cluster RCT | 180 | 75.3 (8.8) | 66.7% | Living in low-income state and federally funded housing developments | 62.2% White  32.2% Black/African  4.4% Hispanic/Latino  2.2% Asian American  3.3% Other/Refused/Unknown |
| Lo et al, 2020 | Boston, USA | Qualitative;  Focus groups  1:1 telephone interviews | 41 | 78.3(10.4) | 68.3% | Living in low-income state and federally funded housing developments | 82.9% White 7.3% Black/African American 4.9% Asian 4.9% Other |
| Manson et al, 2017 | Toronto, Canada | Qualitative; Focus groups | 87 | 20.7% 55-64 40.2% 65-74 39.1% 75+ | 60.9% | - Education: 45.9% <High school 36.8% High school 17.3% >High school - Annual income: 67.9% <Can$14,000 22.9% $14,000 -30,000 9.2% >$30,000 | 42.5% South American 25.3% European  18.4% Chinese  9.2% South Asian  3.5% Caribbean 1.1% Other |
| Moore-Harrison et al, 2008 | Athens, Georgia, USA | RCT | 26 | 71.5(8.1) | 84.6% | - Annual income: 80.8% ≤$20,000 38% <$9,750 = below poverty level - Education:   61.5% ≤12 years | 34.6% African American |
| Owusu et al, 2022 | Ohio, USA | RCT | 213 | 71.85 (5.88) | 100% | 44% Disadvantaged (≤high school education and/or a median household income of ≤$35,000) | 56% Non-Hispanic White 44% African American |
| Patch et al, 2021 | San Diego, USA | Mixed Methods;  Cluster RCT  Focus groups | 60 | 75 (9.45) | 84% | - Living in low-income senior housing complexes - Annual household income: 72% <$20,000 22% $20,000-$39,999 6% ≥$40,000 - Highest education completed: 5% Junior or less 17% some high school 50% some college or vocational 17% completed college 12% completed graduate degree | 70% non-Hispanic white |
| Prins et al, 2019 | Rotterdam, The Netherlands | Non-randomised | 639 | 67.5 (9.2) | 46% | - Living in socioeconomically deprived neighbourhoods - Educational level: None:   Physical condition: 9.3%  Social condition: 6.2%  Combined Physical + Social: 0%  Con: 11.1% Low:  Physical: 51.9%  Social: 29.9% Combined: 41.2% Con: 49.4% Middle:  Physical: 31.8%  Social: 35.1% Combined: 46.6% Con: 30.9% High:  Physical: 7%  Social: 28.9% Combined: 12.2% Con: 8.6% | Region of birth:  The Netherlands: Physical: 69.8%  Social: 69.1%  Combined: 93.9%  Con: 59.3%  Western:  Physical: 3.9%  Social: 6.2%  Combined: 1.4%  Con: 0%  Non-Western:  Physical: 26.4%  Social: 24.7%  Combined: 4.7%  Con: 40.7% |
| Rodriguez Espinosa et al, 2023 | San Francisco Bay Area, USA | Qualitative; Ripple Effects Mapping | 35 | 72.14 (7.33) | 82% | - Low-income housing sites - Education: 77% college educated | - PA + Our Voice Session 1: Non-Hispanic White: 72.7% Asian: 18.2% Black: 0% Hispanic/Latino: 9.1% Session 2: Non-Hispanic White: 85.7% Asian: 0% Black: 14.3% Hispanic/Latino: 0% - PA + Health Education Session 3: Non-Hispanic White: 57.1% Asian: 14.3% Black: 14.3% Hispanic/Latino = 14.3% Session 4: Non-Hispanic White: 80% Asian: 10% Black: 10% Hispanic/Latino: 0% |
| Sharpe et al, 1997 | South Carolina, USA | Mixed Methods;  Non-randomised,  Focus groups  Interviews | 110 | Int =73.7 (6.5)  Con = 77.9 (7.8) | Int: 89%  Con: 84% | - Living in areas with high levels of those aged 65+ years living below the poverty level (38%, 26% & 35% in selected areas compared to 15% in county as a whole) - Mean (SD) education, years  Int = 7.9 (2.8) Con = 7.4 (3.7) | Int: 100% Black Con: 71% Black 29% White |
| Stathi et al, 2022 | Bristol & Bath; Birmingham; Devon, UK | RCT | 777 | 77.6 (6.8) | 66% | - IMD quintile  Int: 10% Q1 (most deprived)  20% Q2  22% Q3  20% Q4  28% Q5 (least deprived)   Con: 12% Q1 (most deprived)  20% Q2  19% Q3  20% Q4  29% Q5 (least deprived)   - Highest educational level: Int: 8% <Secondary  35% Completed secondary  29% Some college/vocational  22% College/University degree  8% Graduate degree/higher   Con: 9% <Secondary  42% Completed secondary  24% Some college/vocational  20% College/University degree  5% Graduate degree/higher   - Home ownership Int: 86% own home Con: 83% own home | Int:  94% White 3% African/Caribbean 1% Asian 1% Other/mixed  Con: 96% White 2% African/Caribbean 1% Asian 1% Other/mixed |
| Stewart et al, 1997 | USA | Non-randomised | 89 | 76.9(6.9) | 83% | - Living in low-income congregate housing facilities - Mean (SD) years of education = 13.8(3.5) | 9% minorities |
| Stewart et al, 2006 | San Francisco, USA | Mixed Methods;  Pre-post design,  Discussion group | 321 | Network site: 5% <60  71% 60-74  21% 75-84  3% >85   30th Street site: 5% <60 44% 60-74  43% 75-84  9% >85   Sequoia site: 1% <60  48% 60-74  44% 75-84  8% >85 | Network site: 97%  30th Street site: 76%  Sequoia site: 87% | - From low-income areas - Education: 30th Street site:   39% ≤6 years  19% >6 years & <high school  20% Completed high school  12% Some college  11% College degree or higher  Sequoia site:  3% ≤6 years  7% >6 years & <high school  21% Completed high school  38% Some college  30% College degree or higher Education not assessed at Network site | Network site:  2% Asian  90% African American  85 White  30^th^ Street site:  3% Asian  4% Filipino  84% Hispanic/Latino  8% White  1% Other  Sequoia site:  9% Asian  9% African American  7% Hispanic/Latino  67% White  9% Other |
| VanRavenstein & Davis, 2018 | Charleston, South Carolina, USA | Qualitative;  Telephone interviews | 12 | Garden Vistas site =72.8(9.7) Garden North site =72.3(7.9) | Garden Vistas site: 83% Garden North site: 93% | Living in low-income older adult housing | Garden Vistas site:  50% White  50% Black Garden North site:  100% Black |
| VanRavenstein et al, 2020 | USA | Mixed Methods;  Pre-post design,  1:1 interviews | 12 | 72.3 | 91.7% | Low-income, living in affordable housing apartments | 100% African American |
| Vieira et al, 2019 | USA | Cluster RCT | 29 | ≥65 years | Not reported | Living in socio-economically disadvantaged neighbourhoods | 100% Hispanic |
| Wang, 2010 | Philadelphia, USA | RCT | 18 | 74.9 (8.4) | 88.9% | - Living in low-income senior housing - Mean (SD) income/month = $952 (319.02) - Mean (SD) years of education = 13.6 (2.7) | 72.2% White 27.8% African American |
| Wang & Glicksman, 2013 | Philadelphia, USA | Qualitative;  Focus groups | 20 | 71.5 | 70.5% | Living in low-income senior housing | 88.2% African American 5.8% Hispanic 5.8% Biracial/Multiracial |
| Yin et al, 2021 | San Antonio, Texas, USA | Non-randomised three arm study | 49 | Int = 74.9 (6.3) Con =73.9 (8.3) | 85.7% | - Low-income using congregate meals program - Education:   <High School Int: 26.5%  Con: 60% | 100% Latino |

Int = Intervention group; Con = control group; IMD = Index of Multiple Deprivation; AMI = area median income; PA = Physical activity

^a^ Study reports this was placed in lowest 21% of income in city of Sao Paulo

^b^Baseline characteristics taken from (1)

References

1. Marsh AP, Lovato LC, Glynn NW, Kennedy K, Castro C, Domanchuk K, et al. Lifestyle interventions and independence for elders study: recruitment and baseline characteristics. Journals of Gerontology Series A: Biomedical Sciences and Medical Sciences. 2013;68(12):1549-58.
