# Supplementary material for: The effectiveness and acceptability of physical activity interventions amongst older adults with lower socioeconomic status: a mixed methods systematic review
Source: Int J Behav Nutr Phys Act. 2024 Oct 22;21:121. doi: 10.1186/s12966-024-01666-8 (PMC11495005; doi:10.1186/s12966-024-01666-8)
Supplement: Supplementary file 4 — Additional file 4: Quality assessment of studies using Mixed Methods Appraisal Tool (MMAT), version 2018 criteria [file 12966_2024_1666_MOESM4_ESM.docx]

**Additional file 4. Quality assessment of studies using Mixed Methods Appraisal Tool (MMAT), version 2018 criteria**

| **Author, year** | **Type of study** | **Qualitative studies** | | | | | **Quantitative studies** | | | | | | | | | | **Mixed Methods studies** | | | | |
| --- | --- | --- | --- | --- | --- | --- | --- | --- | --- | --- | --- | --- | --- | --- | --- | --- | --- | --- | --- | --- | --- |
|  |  |  | | | | | **Randomised Controlled Trials** | | | | | **Non-Randomised** | | | | |  | | | | |
|  |  | **1.1** | **1.2** | **1.3** | **1.4** | **1.5** | **2.1** | **2.2** | **2.3** | **2.4** | **2.5** | **3.1** | **3.2** | **3.3** | **3.4** | **3.5** | **5.1** | **5.2** | **5.3** | **5.4** | **5.5** |
| Almeida et al, 2013 | RCT |  | | | | | CT | Y | N | CT | N |  | | | | |  | | | | |
| Bann et al, 2016 | RCT |  | | | | | Y | Y | Y | Y | N |  | | | | |  | | | | |
| Batik et al, 2008 | RCT |  | | | | | CT | Y | N | CT | CT |  | | | | |  | | | | |
| Brandao et al, 2021 | RCT |  | | | | | Y | Y | Y | Y | Y |  | | | | |  | | | | |
| Britten et al, 2023 | Mixed Methods | Y | Y | Y | Y | Y |  | | | | | CT | Y | N | N | N | Y | N | N | Y | N |
| Crist et al, 2022 | RCT |  | | | | | Y | N | Y | N | N |  | | | | |  | | | | |
| Cwirlej-Sozanska et al, 2018 | RCT |  | | | | | Y | Y | Y | Y | CT |  | | | | |  | | | | |
| Evans et al, 2018 | Qualitative | Y | Y | Y | Y | Y |  | | | | |  | | | | |  | | | | |
| Hammerback et al, 2012 | Mixed Methods | Y | Y | CT | CT | CT |  | | | | | Y | Y | N | Y | N | Y | Y | Y | Y | N |
| King et al, 2013 | RCT |  | | | | | Y | Y | Y | N | Y |  | | | | |  | | | | |
| Kolbe-Alexander et al, 2006 | RCT |  | | | | | Y | N | CT | N | CT |  | | | | |  | | | | |
| Lee et al, 2016 | Non-Randomised |  | | | | |  | | | | | CT | Y | CT | N | CT |  | | | | |
| Lipsitz et al, 2019 | RCT |  | | | | | Y | Y | N | Y | N |  | | | | |  | | | | |
| Lo et al, 2020 | Qualitative | Y | Y | Y | Y | Y |  | | | | |  | | | | |  | | | | |
| Manson et al, 2017 | Qualitative | Y | Y | Y | Y | Y |  | | | | |  | | | | |  | | | | |
| Moore-Harrison et al, 2008 | RCT |  | | | | | CT | Y | Y | N | Y |  | | | | |  | | | | |
| Owusu et al, 2022 | RCT |  | | | | | Y | Y | Y | CT | Y |  | | | | |  | | | | |
| Patch et al, 2021 | Mixed Methods | Y | CT | CT | CT | CT | Y | Y | Y | N | Y |  | | | | | Y | Y | Y | Y | N |
| Prins et al, 2019 | Non-Randomised |  | | | | |  | | | | | Y | Y | N | Y | N |  | | | | |
| Rodriguez Espinosa et al, 2023 | Qualitative | Y | Y | Y | Y | Y |  | | | | |  | | | | |  | | | | |
| Sharpe et al, 1997 | Mixed Methods | Y | CT | CT | CT | CT |  | | | | | Y | Y | Y | Y | N | N | Y | Y | Y | N |
| Stathi et al, 2022 | RCT |  | | | | | Y | Y | Y | Y | N |  | | | | |  | | | | |
| Stewart et al, 1997 | Non-Randomised |  | | | | |  | | | | | Y | Y | Y | Y | N |  | | | | |
| Stewart et al, 2006 | Mixed Methods | Y | CT | CT | CT | CT |  | | | | | N | Y | N | N | N | CT | Y | Y | Y | N |
| VanRavenstein & Davis, 2018 | Qualitative | CT | CT | CT | Y | Y |  | | | | |  | | | | |  | | | | |
| VanRavenstein et al, 2020 | Mixed Methods | Y | Y | CT | Y | CT |  | | | | | N | Y | Y | N | Y | Y | Y | N | CT | N |
| Vieira et al, 2019 | RCT |  | | | | | CT | CT | CT | CT | CT |  | | | | |  | | | | |
| Wang, 2010 | RCT |  | | | | | CT | Y | Y | CT | N |  | | | | |  | | | | |
| Wang & Glicksman, 2013 | Qualitative | Y | Y | CT | Y | Y |  | | | | |  | | | | |  | | | | |
| Yin et al, 2021 | Non-Randomised |  | | | | |  | | | | | Y | Y | Y | Y | N |  | | | | |

Y = Yes; N = No; CT = Can’t tell; RCT = Randomised Controlled Trial

1.1 = Is the qualitative approach appropriate to answer the research question?; 1.2 = Are the qualitative data collection methods adequate to address the research question?; 1.3 = Are the findings adequately derived from the data?; 1.4 = Is the interpretation of results sufficiently substantiated by data?; 1.5 = Is there coherence between qualitative data sources, collection, analysis and interpretation?; 2.1 = Is randomization appropriately performed?; 2.2 = Are the groups comparable at baseline?; 2.3 = Are there complete outcome data?; 2.4 = Are outcome assessors blinded to the intervention provided?; 2.5 = Did the participants adhere to the assigned intervention?; 3.1 = Are the participants representative of the target population?; 3.2 = Are measurements appropriate regarding both the outcome and intervention (or exposure)?; 3.3 = Are there complete outcome data?; 3.4 = Are the confounders accounted for in the design and analysis?; 3.5 = During the study period, is the intervention (or exposure) administered as intended?; 5.1 = Is there an adequate rationale for using a mixed methods design to address the research question?; 5.2 = Are the different components of the study effectively integrated to answer the research question?; 5.3 = Are the outputs of the integration of qualitative and quantitative components adequately interpreted?; 5.4 = Are divergences and inconsistencies between quantitative and qualitative results adequately addressed?; 5.5 = Do the different components of the study adhere to the quality criteria of each tradition of the methods involved?
