# Supplementary material for: The effectiveness and acceptability of physical activity interventions amongst older adults with lower socioeconomic status: a mixed methods systematic review
Source: Int J Behav Nutr Phys Act. 2024 Oct 22;21:121. doi: 10.1186/s12966-024-01666-8 (PMC11495005; doi:10.1186/s12966-024-01666-8)
Supplement: Supplementary file 5 — Additional file 5: Characteristics of included interventions, study outcomes and which review question each addressed [file 12966_2024_1666_MOESM5_ESM.docx]

**Additional file 5. Characteristics of included interventions, study outcomes and which review question each addressed.**

| **Study** | **Setting** | **Type of PA** | **Mode of delivery** | **Theoretical framework / BCTs** | **Duration** | **Comparator**  (if applicable) | **Quantitative Outcomes**  (if applicable) | **Review question addressed** |
| --- | --- | --- | --- | --- | --- | --- | --- | --- |
| Almeida et al, 2013 | Clinical (hospital centre) + At home | Strength & balance | - 3 x 50 mins per week - Fully supervised: face to face supervised sessions - Minimally supervised: 1 x supervised session every 2 weeks + exercises at home 3x per week | Not reported | 4 months | No intervention | - 400MWT - BBS - TUG - Tandem Walk Test - STS - LOS | Effectiveness |
| Bann et al, 2016^a^ | Clinical centres + At home | Walking + Strength & balance | - Goal of 150 mind walking/week - 2x sessions at centre per week + instructional materials to reinforce strength training at home - 3x 10 mins leg strengthening with ankle weights per week | Based on Social Cognitive Theory and Transtheoretical Model | 2.6 years on average (followed up every 6 months) | Successful Ageing Health educational programme on topics excluding physical activity | - Mobility disability (400MWT) | Effectiveness |
| Batik et al, 2008 | Community + At home | Participant choice (combination of home & community programs) | - Motivational support via telephone from older adult volunteers | Incorporated principles of self-efficacy and tailoring to support individual’s readiness to change | 6 months minimum | Received guide to local activity resources, handouts on benefits of exercise, tips on safety and strength & balance | - Self-reported PA (RAPA) - HbA1c | Effectiveness |
| Brandao et al, 2021 | At home | Aerobic + strength & balance | - 3x 40 mins sessions per week minimum - 2-3 sets of 5-15 repetitions for each exercise - Target stress rate of 13-15 on Borg perceived exertion scale - Sessions unsupervised but participants received home visits every 15 days for further guidance and encouragement - Educational booklet with illustrations of exercises and diary to record weekly activities - Guidance given to family members to help/encourage | Not reported | 12 weeks | Educational booklet with information related to ADLs (food, hygiene, sleep) | - TUG - QoL (WHOQOL-OLD) | Effectiveness |
| Britten et al, 2023^b^ | Community centres | Dance | - 1 x 1 hour group sessions per week - Delivered by dance instructors | Not reported | 12 months | No control group | N/A | Acceptability |
| Crist et al, 2022 | Community centres | Walking | - 2x group walks per week - Delivered by volunteer peer health coaches (PHCs) - Participants encouraged to gradually increase steps by 2000 per day from individual baseline | - Combined components of Empowerment Theory and Social Cognitive Theory - Used Socioecological Model as framework - BCTs included goal setting and self-monitoring | 2 years | Received five wellness presentations unrelated to physical activity | - Objective PA (accelerometer) - Blood pressure - 6MWT - QoL (PQoL-20) - Depression (CES-D-10) | Effectiveness |
| Cwirlej-Sozanska et al, 2018 | University | Aerobic + strength & balance | - 2x 60 mins sessions per week - Delivered by two physiotherapists | Not reported | 16 weeks | 7 x 45 mins health education sessions on PA & nutrition + 1 session on oral health | - TUG - FRT - Tandem Stance, Walk & Pivot - SFT | Effectiveness |
| Evans et al, 2018 | Bingo Club | Chair-based exercise, dancercise, and line dancing | - 3x 20-30 mins sessions per week led by exercise instructors - Included health educational messages | Educational messages informed by Socioecological Model & Transtheoretical Model | 12 weeks | N/A | N/A | Acceptability |
| Hammerback et al, 2012^b^ | Telephone-based | Not specified | - 1:1 telephone support delivered by volunteers trained in motivational interviewing - 2x 20-30 mins telephone calls per month for first 6 months, 1x per month for up to an additional 6 months | Not reported | 6 months | No control group | N/A | Acceptability |
| King et al, 2013 | Community centre | Walking | - Virtual advisor sessions - Participants encouraged to interact weekly or whenever visiting community centre | - Based on Social Cognitive Theory and Transtheoretical Model - BCTs included: Self-assessment,   motivationally tailored goal setting,  individualised feedback,  positive reinforcement and knowledge enhancement related to benefits of physically active lifestyle | 4 months | Monthly 1 hour health education sessions in non-PA topics including nutrition and home safety | - Walking -Self-report (CHAMPS walking items)   Objective (Pedometer)^c^   - Acceptability (6-item computer credibility scale,   12-item bonding subscale of Working Alliance Inventory,  4-item Computer Attitude Scale)   - Motivation Motivational Processes of Change inventory) | Effectiveness  Acceptability |
| Kolbe-Alexander et al, 2006 | Community centres | Strength & balance | - 2x 45-50 mins group sessions per week - 2x 10-15 repetitions for each exercise - Researcher conducted sessions | Not reported | 20 weeks | 2x weekly 1 hour relaxation classes or arts/crafts and sing-along activities | - Self-report PA (YPAS) - ADL & IADL questionnaire - Self-reported health status - STS - Grip strength (hand-held dynamometer) - 6MWT - 20MWT - Static balance test - Dynamic balance (tandem walk) - Blood pressure - BMI | Effectiveness |
| Lee et al, 2016 | Community centres | Yoga; Dance | - 2x 60 mins per week - Intensity higher than medium depending on individual's exercise capability; safe range was set based on level 11-13 RPE | Not reported | 36 weeks | Sports massage^d^ | - Lower extremity muscle strength/endurance (Chair stand test) - Upper body muscle strength/endurance (Arm curl test) - Muscular strength/endurance (Modified sit and reach test) - Upper body flexibility (Back scratch test) - Agility (TUG) | Effectiveness |
| Lipsitz et al, 2019 | Community room at public housing facilities | Tai Chi | - 2x group classes per week led by experienced tai chi instructors - Given DVDs, illustrated manuals and instructions for home practice on non-class days - Minimum 20mins home practice on 3 non-class days/week - Monthly social calls by research assistant | Not reported | 12 months (looked at 6 month change) | - Monthly health educational group sessions: 30 mins lecture + 30 mins group discussion - Monthly social calls by research assistant | - Self-reported PA (PASE) - SPPB - Gait speed - Falls - Balance confidence (ABC) - HRQoL (SF-12) - Depression (20Q CES-D) | Effectiveness |
| Lo et al, 2020 | Community room at public housing facilities | Tai Chi | - 2x group classes per week led by experienced tai chi instructors - Given DVDs, illustrated manuals and instructions for home practice on non-class days - Minimum 20mins home practice on 3 non-class days/week - Monthly social calls by research assistant | Not reported | 12 months | N/A | N/A | Acceptability |
| Manson et al, 2017 | Community | Tai Chi | Not reported | Not reported | 16 weeks | N/A | N/A | Acceptability |
| Moore-Harrison et al, 2008 | Older adult public housing apartment complex | Walking + strength & balance | - 3x week classes at 60-75% heart rate maximum, RPE 12-14 (moderate intensity) | Not reported | 16 weeks | Nutrition education:  Given presentations about benefits of nutrition focusing on fruit and vegetables every 2 weeks | - SF-36 - CS-PFP-10 - Peak Aerobic Capacity (VO2peak) - Lower Limb Extension Strength (1RPM leg press machine) | Effectiveness |
| Owusu et al, 2022 | Community centre | Aerobic (treadmill/elliptical/stationary bike) + Resistance | - 3x 60 mins group sessions per week (30mins aerobic + 30mins resistance) - Supervised by certified exercise trainer for first 20 weeks then unsupervised for 32 weeks - Moderate intensity: Aerobic performed at 50-70% of maximum heart rate and Resistance exercises 1-2 sets of 10 reps, gradually increased to 3 sets by session 5 | Not reported | 20 weeks | - 1x 60 mins support group session per week looking at impact of cancer diagnosis on participant's lives, family dynamics & ageing. - Also included   activities (e.g. guided meditation, arts, journalling & nutrition, drumming, cooking class) | - Objective PA (FitBit) - Self-report PA (MLTPAQ) - SPPB - 6MWT - ADLs/IADLs - SF-36 physical function - 4 metre gait speed - Risk of functional decline (VES) - HRQoL (FACT-B) - Sleep (PSQI) | Effectiveness |
| Patch et al, 2021 | Low-income senior housing | Walking | 1x 1hr group meeting per week (group walk + education about physical activity) | - Goal setting - Social support - Positive thinking for increasing PA | 8 weeks | Advocacy program: training to increase advocacy skills and self-efficacy.^e^ | - Objective PA (accelerometer) - Self-report PA (IPAQ; BRFSS) | Effectiveness |
| Prins et al, 2019 | Within neighbourhoods (not otherwise specified) | Walking | 3 conditions:   - Physical intervention: designated walking routes (approx 5km) - Social intervention: peer-led neighbourhood walking groups - Combined physical + social intervention | Not reported | 9 months | No intervention | - Self-report walking (IPAQ) | Effectiveness |
| Rodriguez Espinosa et al, 2023 | Low-income senior housing sites | Not reported | - 12x 90min semi-monthly group sessions onsite for 6 months - Delivered by trained research staff - For second 6 months participants received either healthy ageing education for or "Our Voice" where participants were encouraged to become ‘citizen scientists’ and document/improve features of their local communities which promote/hinder healthy living | Based on Socioecological Model | 12 months | N/A | N/A | Acceptability |
| Sharpe et al, 1997 | Congregate meal centres | Strength & balance | - Low intensity program - movements performed sitting & standing - Handheld weights (after week 6) & dance movements (after week 16) also included - Instructor-led classes 2x per week   + Manual with instructions/pictures to perform movements at home   - Peer leaders trained to lead exercise classes at end of 1 year - First 3 weeks: 4-6 reps of each movement; Next 12 weeks: 6-20 reps; Rest of year: 12-20 reps | - Incentives for attendance - Goal setting during week 2 - Progress assessment against goals at six months | 1 year | No intervention | - Mobility (balance & gait; POMA short form) - Grip strength (hand dynamometer) - Tandem balance - One-leg balance - Timed walk (normal speed & maximum speed) - Perceived change in physical functioning | Effectiveness  Acceptability |
| Stathi et al, 2022 | Leisure & community centres | Strength & balance | - Group exercise sessions delivered by qualified exercise professionals - Behavioural maintenance programme started after 9 weeks - 45 min 1x per week - provided PA/health info - reduced to 1x per month from week 25 - 2x 1hour sessions per week for 12 weeks, 1x per week for 40 weeks - Moderate-vigorous intensity (11-16 RPE) strength-based exercises - Light-moderate intensity games-based activities (8-13 RPE) - Individualised approach participants progressed at own pace | - Derived from Social Cognitive Theory; Self-Determination Theory; Skills for Maintenance Model   BCTs included:   - Building intrinsic motivation - Making realistic plans for sustainable activity - Pre-empting/overcoming barriers - Maximising enjoyment, social interaction and group identity - Engaging external social support - Using self-monitoring & self-regulatory techniques | 12 months | 3x 60-90 mins workshops covering healthy ageing topics with no PA content delivered before 6, 12 & 24 months assessments | - SPPB^f^ | Effectiveness |
| Stewart et al, 1997 | Community-based | Participant choice | - Participants helped to select activities available within their community using a directory which provided info on moderate-level PA classes/programs - Participants encouraged to aim for target of 3-5x per week - Written materials & monthly newsletter provided info/tips for starting out and maintaining activities - Monthly group meetings - Telephone support from staff | - Derived from Social Cognitive Theory   BCTs included:   - Self-monitoring (Activity logs) - Emphasising health benefits of PA - Recognising/overcoming barriers - Support/encouragement from staff - Incentives | 6 months | Wait-list control | - Self-report PA - HRQoL | Effectiveness |
| Stewart et al, 2006^b^ | Senior centres | Participant choice | - Group workshops & PA directories at all three sites - Site 1 (Network) provided newsletters and added weekly program-based exercise class - offered 7/8 exercises & health-related workshops and weekly exercise class - Site 2 (30th Street) used program bulletin board - offered 6 exercise-related workshops, 2/3 additional exercises & health-related presentations, 5-7 Q&A forums with physician about exercise, 1-4 special celebration events - Site 3 (Sequoia) offered 8 workshops and celebration at completion of program | Not reported | 6 months | No control group | N/A | Acceptability |
| VanRavenstein & Davis, 2018 | Low-income older adult housing sites | Strength & balance (Otago exercise program) | - 2x weekly Televideo group classes delivered by physical therapy student - Participants also wore Fitbits to track own activity | Not reported | 12 weeks | N/A | N/A | Acceptability |
| VanRavenstein et al, 2020^b^ | Affordable public housing apartments | Strength & balance (Otago exercise program) | - 2x exercise sessions per week + Independent walking 3x per week - 2 conditions: - On-site instructor led group sessions - Telehealth instructor-led sessions | Not reported | 12 weeks | No control group | N/A | Acceptability |
| Vieira et al, 2019 | Senior centres | Strength & balance (Otago exercise program) | - 2x Group sessions (30min group exercise, 30min walking  + 30 min group nutrition sessions) per week - Led by physical therapy students | Not reported | 3 months | No intervention | - Body composition - Grip strength - STS - Blood pressure - HbA1c | Effectiveness |
| Wang, 2010 | Low-income senior housing site | Yoga | - 2x 1hr In-person group sessions per week (45min posturing & breathing, 15min of relaxation & teachings) | Not reported | 4 weeks | - Socialization group: 2x 1hr sessions per week shown films - Asked to continue with daily routines/activities & not participate in yoga classes | - 1-leg stand - STS - Sit-and-reach test - Depression (CES-D) - Morale (PGCMS revised) - Hope (HHI) - Social isolation (UCLA Loneliness Scale) | Effectiveness |
| Wang & Glicksman, 2013 | Low-income senior housing sites | Gardening | Not reported | Not reported | Not reported | N/A | N/A | Acceptability |
| Yin et al, 2021 | Senior centres + At home | Qigong (Five Animal Play) | - In-person group sessions led by community health worker (changed to weekly individual phone calls after week 4 due to COVID) - At home practice using tablet videos - Text reminders 2x per week re. home practice | - Based on Social Cognitive Theory - BCTs included:   Vicarious experience Goal setting Reinforcement Role modelling Social support Self-regulation | 16 weeks | - Healthy ageing program delivered by community health workers - Content included exercise, nutrition, finances, advance care planning, community engagement, healthy relationships | - Satisfaction with program & services (CSQ-8) - ADL/IADL (FSQ) - QoL (SF-12) - Chronic pain (9-item BPI) | Effectiveness  Acceptability |

^a^ Some details taken from (1)

^b^ Mixed methods studies with no control group whose quantitative outcomes/findings were not included in data synthesis looking at effectiveness

^c^ Not measured in the control group only intervention group therefore not included in data synthesis

^d^ For the purposes of this review this group was used as the comparator as this was not deemed to be a physical activity intervention according to review criteria

^e^ This condition was the intervention group and the physical activity condition was the control group in this study, but for the purposes of this review we focused on the physical activity condition

^f^ Other outcomes were measured but this was the only one stratified by SES and as such the only outcome for which results for low SES older adults could be extracted

400MWT = 400-Meter Walk Test; BBS = Berg Balance Scale; TUG = Timed Up and Go; SST = Sit-to-Stand Test; LOS = Limits of Stability Test; SCT = Social Cognitive Theory; TTM = Transtheoretical Model; Int = Intervention; RAPA = Rapid Assessment of Physical Activity; ADLs = Activities of Daily Living; QoL = Quality of Life; WHOQOL-OLD = World Health Organisation Quality of Life Instrument – older adults module; SALS = Short Active Lives Survey; EQ-VAS = EuroQol Visual Analogue Scale; SEM = Social Ecological Model; 6MWT = 6 Minute Walk Test; PQoL = Perceived Quality of Life; CES-D = Centre for Epidemiological Studies Depression Scale; FRT = Functional Reach Test; SFT = Senior Fitness Test; CHAMPS = Community Health Activities Model Program for Seniors; YPAS = Yale Physical Activity Survey; IADL = Instrumental Activities of Daily Living; PASE = Physical Activity Scale for the Elderly; SPPB = Short Physical Performance Battery; 20MWT = 20-Metre Walk Test; ABC = Activities-specific Balance Confidence Scale; HRQoL = Health-related Quality of Life; RPE = Rate of Perceived Exertion; SF= Short Forms Health Survey; CSPFP = Continuous Scale Physical Functional Performance; MLTPAQ = Minnesota Leisure Time Physical Activity Questionnaire; VES = Vulnerable Elders Survey; FACT-B = Functional Assessment of Cancer Therapy-Breast Cancer; PSQI = Pittsburgh Sleep Quality Index; BRFSS = Behavioural Risk Factor Surveillance System; IPAQ = International Physical Activity Questionnaire; POMA = Performance Oriented Mobility Assessment; MSEQ = Muscle Strengthening Exercise Questionnaire; MAT = Mobility Assessment Tool; SCI = Sleep Condition Indicator; 2MWT = 2-Minute Walk Test; SEE = Self Efficacy for Exercise Scale; LSNS = Lubben Social Network Scale; PGCMS = Philadelphia Geriatric Centre Morale Scale; HHI = Herth Hope Index; CSQ = Client Satisfaction Questionnaire; BPI = Brief Pain Inventory

References

1. Fielding RA, Rejeski WJ, Blair S, Church T, Espeland MA, Gill TM, et al. The lifestyle interventions and independence for elders study: design and methods. Journals of Gerontology Series A: Biomedical Sciences and Medical Sciences. 2011;66(11):1226-37.
