# Supplementary material for: The effectiveness and acceptability of physical activity interventions amongst older adults with lower socioeconomic status: a mixed methods systematic review
Source: Int J Behav Nutr Phys Act. 2024 Oct 22;21:121. doi: 10.1186/s12966-024-01666-8 (PMC11495005; doi:10.1186/s12966-024-01666-8)
Supplement: Supplementary file 6 — Additional file 6: Quantitative data from studies included in systematic review synthesis [file 12966_2024_1666_MOESM6_ESM.docx]

**Additional file 6. Quantitative data from studies included in systematic review synthesis**

| **Primary outcome: Physical Activity** | | | | | | **Intervention** | | | **Control** | | |  |
| --- | --- | --- | --- | --- | --- | --- | --- | --- | --- | --- | --- | --- |
| **Study (measure)** | **Intervention (n)** | **Control (n)** | | **Timepoint of follow up** | **Main results** | **Baseline mean (SD)** | **F/U mean (SD)** | **Other data reported** | **Baseline mean (SD)** | **F/U mean (SD)** | **Other data reported** |  |
| **Objectively measured PA n=3 studies** | | | | | | | | | | | |  |
| *Moderate to Vigorous Physical Activity (MVPA) n=2* | | | | | | | | | | | |  |
| Crist 2022 (MVPA min/day) | 267 | | 209 | 24 months | Both low- and higher-income  intervention participants increased MVPA compared to  their respective control condition.  Low-income Int. had similar increase in MVPA to high income Int, but had greater decrease from 12-24 months. | 11.7 (12.8) | NR |  | 22.1 (23) | NR |  |  |
| Patch 2021 (MVPA min/day) | 32 | | 17 | 8 weeks | No sig difference (p = .481) | 17.4 (19.2) | 17.6 (22.4) |  | 5.2 (9) | 3.6 (6.5) |  |  |
| *Daily steps n=1* | | | | | | | | | | | |  |
| Owusu 2022 (daily steps) | 86 in total - unable to know how many in each arm due to missing data | | | 20 weeks | No sig difference (p=0.93) | NR | NR | Estimate of change from baseline (int. vs. control): -31.224, SE: 376.388, 95% CI (773.490, 711.042) | NR | NR |  |  |
| **Self-reported PA n=8 studies** | | | | | | | | | | | |  |
| *Total physical activity n=5* | | | | | | | | | | | |  |
| Lipsitz 2019 (PASE total score) | 93 | | 87 | 6 months | No sig difference (p=0.92) | 72.1 (50.4) | NR | Estimate of change from baseline: -3.42, SE: 5.17 | NR | NR | Estimate of change from baseline: -4.14 (SE: 5.37) |  |
| Owusu (MLTPAQ questionnaire) | 77 in total - unable to know how many in each arm due to missing data | | | 20 weeks | No sig difference (p=.7087) | NR | NR | Estimate of change from baseline (int. vs. control): -142.43, SE: 380.69, 95% CI (-898.64, 608.78) | NR | NR |  |  |
| Kolbe-Alexander 2006 (total weekly energy expenditure, and breakdown of exercise related vs recreation) | 2 intervention groups (n=32 and n=27) | | 22 | 20 weeks | EX1 and EX2 increase in total weekly and exercise-related energy expenditure compared to CON (p=.0008)  but no consistent change in total weekly energy expenditure (composite score of work, yard, care-giving, recreation and exercise activities) | NR | NR |  | NR | NR |  |  |
| Stewart 1997 (weekly frequency of all PA/ weekly caloric expenditure of all PA) | 59 | | 30 | 6 months | No difference in frequency of PA or caloric expenditure between Int and Con at 6mths (p>.10) | NR | NR |  | NR | NR |  | |
| Batik 2008 (RAPA score) | 14 | | 170 | ≥ 6 months | Distribution of RAPA scores from baseline to ≥6 months:  1-3 "minimal PA" - 42.9% to 28.6% 4 or 5 "suboptimal but some potential health benefit" - 35.7% to 28.6% 6 or 7 "US Surgeon General's rec for optimal PA" - 21.4% to 42.9% (but not sig. p=.25) | NR | NR |  | NR | NR |  |  |
| *Walking n=3* | | | | | | | | | | | |  |
| King 2013 (walking min/wk) | 20 | | 20 | 4 months | Sig. increase (p = .0008) | 36 (67) | NR | Mean change (SD): 253.5 (248.7). Between-group difference of 226.7, 95% CI = 107.0, 346.4, effect size = 1.2 | 63.8 (160.9) | NR | Mean change (SD): 26.8 (67) |  |
| King 2013 (walking fast or briskly min/wk) | 20 | | 20 | 4 months | Sig. increase (p = .002) | NR | NR | Mean change (SD): 147 (167.1), effect size = 1.1 | NR | NR | Mean change (SD): 12.6 (33.3) |  |
| King (walking leisurely min/wk) | 20 | | 20 | 4 months | Not significant (p = .09) | NR | NR | Mean change (SD): 106.5 (211.5), effect size = 0.6 | 63.8 (160.9) | NR | Mean change (SD): 14.2 (51.4) |  |
| Prins 2019 (total walking min/wk) | 3 intervention groups (Physical: n=215, Social n = 130; Combined: n = 181) | | 113 | 9 months | Physical and social conditions had sig increase between baseline and first follow-up (3 months) this was twice as large as control group. The IRR for physical was 1.46 (95%CI: 1.06–2.05) and 1.52 (95%CI:1.07–2.16) for social.  Sig increase in total  walking between baseline and the second follow-up (9 months) in physical condition, 1.4 times greater than in the control condition (IRR: 1.42; 95%CI:1.02–1.99). | Median (95% CI) Physical = 330 (249;409)  Social = 242 (138;346)  Combined = 263 (204;322) | Median (95% CI) Physical = 373 (275;471)  Social = 355 (243;466) Combined = 280 (205;354) | IRR (95% CI)  Physical = 1.42 (1.02;1.99)  Social = 1.42 (0.96;2.10)  Combined = 1.17 (0.83;1.68) | Median (95% CI) 338 (208;468) | Median (95% CI): 383 (228;538) |  |  |
| Prins 2019 (recreational walking min/wk) | 3 intervention groups (Physical: n=215, Social n = 130; Combined: n = 181) | | 113 | 9 months | No sig differences for any group at 3 months and 9 months (p>0.05) | Median (95% CI) Physical = 78 (26;129)  Social = 57 (22;91)  Combined = 116 (82; 149) | Median (95% CI) Physical = 102 (40;163)  Social = 74 (18;130) Combined = 06 (58;154) | IRR (95% CI)  Physical = 0.63 (0.89; 2.89)  Social = 1.48 (0.84; 2.64)  Combined = 1.22 (0.64;2.18) | Median (95% CI) 98 (26;167) | Median (95% CI) 74 (−11;157) |  |  |
| Prins 2019 (utlitarian walking min/wk) | 3 intervention groups (Physical: n=215, Social n = 130; Combined: n = 181) | | 113 | 9 months | Compared to  the control condition, the increase between baseline and  the first follow-up (3 months) was higher (IRR 1.60; 95%CI: 1.06–  2.41) in the physical intervention condition and in the  social intervention condition (IRR: 1.62; 95%CI:1.08–  2.44).  No sig. effect at 9 months for either intervention groups (p>0.05) | Median (95% CI)  Physical = 179 (129;228)  Social = 150 (87;213)  Combined = 104 (60;147) | Median (95%CI) Physical = 207 (132;280)  Social = 186 (104;267) Combined = 122 (85;158) | IRR (95% CI)  Physical = 1.21 (0.83;1.79)  Social = 1.27 (0.87;1.88)  Combined = 1.26 (0.86;1.86) | Median (95% CI)  193 (125;260) | Median (95% CI)  244 (142; 345) |  |  |
| Patch 2021 (transportation PA min/wk) | 32 | | 17 | 8 weeks | Sig. increase in walking for transportation in PA group vs. advocacy group who had small decline (F=4.35, p=.042) | 234.5 (252.6) | 377.5 (317.6) |  | 214.1 (252.2) | 164.4 (207.8) |  |  |
| Patch 2021 (leisure PA min/wk) | 32 | | 17 | 8 weeks | No sig difference (p =.306) | 214.9 (205.6) | 252.5 (283.2) |  | 116.5 (220.5) | 79.1 (89.6) |  |  |
| *Self-report strength n=1* | | | | | | | | | | | |  |
| Patch 2021 (strength training min/wk) | 32 | | 17 | 8 weeks | No sig difference (p =.781) | 93.9 (116.2) | 108.9 (111) |  | 52 (86.3) | 75.9 (130.6) |  |  |
| Patch 2021 (flexibility training min/wk) | 32 | | 17 | 8 weeks | No sig difference (p=.936) | 95.9 (93.2) | 138.3 (134.4) |  | 47.1 (53.6) | 92.6 (140.3) |  |  |

NR = not reported
