# Supplementary material for: The effectiveness and acceptability of physical activity interventions amongst older adults with lower socioeconomic status: a mixed methods systematic review
Source: Int J Behav Nutr Phys Act. 2024 Oct 22;21:121. doi: 10.1186/s12966-024-01666-8 (PMC11495005; doi:10.1186/s12966-024-01666-8)
Supplement: Supplementary file 7 — Additional file 7: Quantitative data for secondary outcomes from studies included in systematic review synthesis [file 12966_2024_1666_MOESM7_ESM.docx]

**Additional file 7. Quantitative data for secondary outcomes from studies included in systematic review synthesis**

| **Secondary outcomes** | | | | | | **Intervention** | | | **Control** | | | |  |
| --- | --- | --- | --- | --- | --- | --- | --- | --- | --- | --- | --- | --- | --- |
| **Study (measure)** | **Intervention (n)** | | **Control (n)** | **Timepoint of follow up** | **Main results** | **Baseline mean (SD)** | **F/U mean (SD)** | **Other data reported** | **Baseline mean (SD)** | **F/U mean (SD)** | | **Other data reported** |  |
| **Physical function measures n=16 studies** | | | | | | | | | | | | |  |
| **Strength n=8 studies** | | | | | | | | | | | | |  |
| *Lower body n=7* | | | | | | | | | | | | |  |
| Almeida 2013 (Sit-to-Stand - weight transfer time sec) | 2 intervention groups (FS: n = 28 and MS: n=22) | 26 | | 4 months | Sig decrease FS group (p =.04); No sig effect for MS group (p=.11); No changes in control group (p=.38) | FS = 0.53 (0.51); MS = 0.58 (0.38) | FS = 0.41 (021); MS = 0.45 (0.28) |  | 0.5 (0.33) | 0.43 (0.26) | |  |  |
| Kolbe-Alexander 2006 (Sit-to-Stand no. of repetitions) | 2 intervention groups (EX1 : n = 32 and EX2: n=27) | 22 | | 20 weeks | Sig. increase in EX1 at 20 weeks only (p<.0001) and EX2 at both 10 weeks and 20 weeks (p<.0001); No changes in Control group | NR | NR |  | NR | NR | |  |  |
| Vieira 2021 (Sit-to-Stand, no. of repetitions) | 8 | 15 | | 3 months | Sig. increase (p=0.025) | 9 (4) | 10 (5) |  | 7 (4) | 7(4) | |  |  |
| Wang 2010 (Sit-to-Stand, seconds) | 5 | 9 | | 4 weeks | No sig difference for yoga (p=.085) or control socialisation group (p=.051) | NR | NR | Mean difference (SD): 6.66 (6.55) | NR | NR | | Mean difference (SD): -10.36 (13.55) |  |
| Lee 2016 (Sit-to-Stand, repetitions) | 2 intervention groups yoga: n=20 and dance: n=20) | 20 | | 36 weeks | No sig time x group effect (F = 0.694, p>.05) | yoga = 14.92 (4.90); dance = 11.00 (4.35) | yoga = 15.23 (2.91); dance = 11.40 (3.64) |  | 8.5 (2.38) | 11.25 (4.57) | |  |  |
| Moore-Harrison 2008 (1RM leg press) | 12 | 12 | | 16 weeks | Both groups demonstrated nonsignificant improvements (7% control group and 6.6% walking exercise group) in 1RM. | NR | NR |  | NR | NR | |  |  |
| Owusu 2022 (leg press) | 60 in total- unable to know how many in each arm due to missing data | | | 20 weeks | Sig. difference (p=0.0014) | NR | NR | Estimate of change from baseline (int. vs. control): 27.706, SE: 28.497, 95% CI (10.917, 44.494) | NR | NR | |  |  |
| *Upper body n=5* | | | | | | | | | | | | |  |
| Kolbe-Alexander 2006 (grip strength, handheld dynamometer kg of isometric force) | 2 intervention groups (EX1:n = 32 and EX2:n=27) | 22 | | 10 and 20 weeks | No sig. difference in grip strength at 10 or 20 weeks | EX1 = 17.23 (5.08); EX2 = 19.89 (5.66) | EX1 = 19.03 (4.61); EX 2 = 21.69 (5.08) |  | 18.11 (5.71) | | 18.3 (6.03) |  |  |
| Vieira 2021 (grip strength, handheld dynamometer, kg) | 8 | 15 | | 3 months | No sig effect (p=0.474) | 23 (7) | 22 (8) |  | 21 (6) | | 21 (8) |  |  |
| Sharpe 1997 (grip strength, hand dynamometer, kg) | 60 | 47 | | 1 year | Time x group not significant (p=0.22) | 18.38 (6.99) | 18.41 (6.78) | Mean change (adjusted for age and race) = -0.06 | 16.74 (8.3) | | 15.56 (8.58) | Mean change (adjusted for age and race) = -1.08 |  |
| Owusu 2022 (chest press 1RM) | 62 in total - unable to know how many in each arm due to missing data | | | 20 weeks | No sig difference (p=0.501) | NR | NR | Estimate of change from baseline (int. vs. control): -2.674, SE: 3.964, 95% CI (-10.506, 5.158) | NR | | NR |  |  |
| Lee 2016 (upper body, right arm curl) | 2 intervention groups yoga: n=20 and dance:n=20) | 20 | | 36 weeks | Sig difference in the time x group mutual effectiveness (F = 3.515, p<.05) | yoga = 22.23 (7.16); dance = 18.80 (6.76) | yoga = 35.61 (12.70); dance = 20.80 (9.31) |  | 11 (8.4) | | 13.75 (1.25) |  |  |
| **Mobility n=6 studies** | | | | | | | | | | | | |  |
| Almeida 2013 (TUG) | 2 intervention groups (FS :n = 28 and MS: n=22) | 26 | | 4 months | Significant improvement in both FS (p < .001) and MS (p < .001) groups | FS = 13.4 (4.2); MS = 15.9 (6.6) | FS = 9.7 (2.4); MS = 12.3 (3.6) |  | 14.2 (4) | 13.6 (3.3) | |  |  |
| Almeida 2013 (400MWT) | 2 intervention groups (FS :n = 28 and MS: n=22) | 26 | | 4 months | Sig decrease in 400m walk time in FS group (p< .001); MS not sig (p=.86); Con not sig (p=.46) | FS = 406.8 (81.4); MS = 452.8 (138.6) | FS = 357.2 (84.7); MS = 448.5 (108.6) |  | 438 (98.2) | 427.5 (117.1) | |  |  |
| Bann 2016 (400MWT) | 230 | 232 | | 2.6 years | Income×intervention interaction terms were not statistically significant for major mobility disability and persistent mobility disability (p=0.90 and 0.79, respectively) | NR | NR |  | NR | NR | |  |  |
| Brandao 2021 (TUG) | 61 | 64 | | 12 weeks | Sig. reduction in time of execution of TUG (*P* < .01) in Int.  Con not sig. (*P* = .7) | 9.1 (2) | 7.1 (1) |  | 9 (2) | 8.7 (2) | |  | |
| Cwirlej-Sozanska 2018 (TUG) | 21 | 23 | | 16 weeks | Sig. difference Int. vs Con., sig decrease in Int. (p<.001) | 11.19 (1.91) | 8.47 (1.69) |  | 10.26 (2.35) | 11.39 (1.92) | |  |  |
| Lee 2016 (TUG) | 2 intervention groups yoga: n=20 and dance: n=20) | 20 | | 36 weeks | No sig time x group effect  F=2.816, p>.05) | yoga = 5.52 (0.85); dance = 8.66 (2.00) | yoga = 8.20 (4.42); dance = 10.05 (2.59) |  | 10.1 (1.68) | 11.64 (2.64) | |  |  |
| Sharpe 1997 (POMA score 0-14) | 59 | 48 | | 1 year | No sig time x group effect p=.10 | 9.81 (1.8) | 9.85 (2.47) | Mean change adjusted for age and race = 0.14 | 9.58 (2.23) | 8.77 (3.23) | | Mean change adjusted for age and race = -0.67 |  |
| **Balance n=4 studies** | | | | | | | | | | | | |  |
| Almeida 2013 (BBS score) | 2 intervention groups (FS: n = 28 and MS: n=22) | 26 | | 4 months | Sig increase in FS (p=.02) No sig change in MS (p=.17) or Con (p=.27) | FS = 53.8 (2.0); MS = 53.3 (3.2) | FS = 54.6 (1.1); MS = 54.0 (1.8) |  | 53.7 (1.8) | 53.4 (2) | |  |  |
| *Dynamic (n=3)* | | | | | | | | | | | | |  |
| Almeida 2013 (Tandem Walk - step width cm) | 2 intervention groups (FS: n = 28 and MS: n=22) | 26 | | 4 months | No sig change for FS group (p =.19) MS (p=.06) or Con (p=.18) | FS = 12.6 (4.4); MS =13.9 (4.6) | FS = 11.4 (4.3); MS = 12.2 (3.7) |  | 13 (5.5) | 11.6 (4.1) | |  |  |
| Almeida 2013 Tandem Walk - speed cm/s) | 2 intervention groups (FS: n = 28 and MS: n=22) | 26 | | 4 months | Sig increase in MS (p=.01) and sig decrease in Con (p=.03); No sig change in FS (p=.30) | FS = 15.8 (6.4); MS = 13.8 (3.5) | FS = 18.5 (5.0); MS =17.2 (5.0) |  | 17.2 (6.9) | 14.5 (5.3) | |  |  |
| Almeida 2013 (Tandem Walk - end sway) | 2 intervention groups (FS: n = 28 and MS: n=22) | 26 | | 4 months | No sig change for FS (p=.08) or Con (.79); sig decrease in MS (p=.02) | FS = 8.3 (3.3); MS = 7.5 (2.3) | FS = 7.0 (2.5); MS = 6.2 (2.0) |  | 7.1 (3.4) | 6.9 (2.6) | |  |  |
| Almeida 2013 (Limits of Stability - movement velocity cm/s) | 2 intervention groups (FS: n = 28 and MS: n=22) | 26 | | 4 months | Sig increase in FS (p<.001) and MS (p=.02); no sig change in Con (p=.97) | FS = 2.83 (0.93); MS = 2.56 (0.86) | FS = 3.71 (1.28); MS = 3.17 (1.28) |  | 2.45 (0.91) | 2.57 (1.09) | |  |  |
| Almeida 2013 (Limits of Stability - maximum excursion cm) | 2 intervention groups (FS: n = 28 and MS: n=22) | 26 | | 4 months | Sig increase in FS (p<.001) and MS (p=.03); no sig change in Con (p=.51) | FS = 70.3 (24.7); MS = 58.5 (19.8) | FS = 74.4 (17.9); MS = 68.3 (19.2) |  | 64.9 (19.6) | 62.8 (16.9) | |  |  |
| Almeida 2013 (Limits of Stability - directional control % of movement) | 2 intervention groups (FS: n = 28 and MS: n=22) | 26 | | 4 months | Sig increase in FS (p=.004) and MS (p=.01); no sig change in Con (p=.65) | FS = 65.2 (16.5); MS = 62.0 (16.2) | FS = 68.4 (13.9); MS = 69.9 (13.3) |  | 67.7 (9) | 63.9 (10.5) | |  |  |
| Cwirlej-Sozanska 2018 (Tandem Walk) | 21 | 23 | | 16 weeks | Sig effect of Int vs Con (p=.001) | 2.95 (1.24) | 4.42 (0.81) |  | 4.13 (0.75) | 4.04 (0.87) | |  |  |
| Cwirlej-Sozanska 2018 (Tandem Pivot) | 21 | 23 | | 16 weeks | Sig effect of Int vs Con (p=.001) | 2.33 (1.27) | 3.85 (1.1) |  | 3.65 (1.07) | 3.34 (0.98) | |  |  |
| Kolbe-Alexander 2006 (Tandem Walk, seconds) | 2 intervention groups (EX1: n = 32 and EX2: n=27) | 22 | | 20 weeks | Sig improvement in EX1 and EX2 groups vs Con (p=0.013) | EX1 = 75.1 (31.3) ; EX2 = 53.3 (17) | EX1 55.3 (13.6) ; EX2 = 37.0 (10.4) |  | 537.7 (29.8) | 52.1 (16.4) | |  |  |
| *Static n=4* | | | | | | | | | | | | |  |
| Kolbe-Alexander 2006 (static balance) | 2 intervention groups (EX1: n = 32 and EX2: n=27) | 22 | | 20 weeks | No sig group x time effect | EX1 = 21.0 (10.2); EX2 = 28.4 (3.8) | EX1 = 28.3 (4.7); EX2 = 28.7 (5.0) |  | 24.9 (7.5) | 28.4 (5.3) | |  |  |
| Wang 2010 (one-leg, seconds) | 4 | 8 | | 4weeks | No sig difference in Int (p=.571) or Con (p=.427) | NR | NR | Mean difference (SD): -0.7 (1.8) | NR | NR | | Mean difference (SD): 0.86 (2.87) |  |
| Sharpe 1997 (tandem balance, seconds) | 35 | 27 | | 1 year | No sig time x group effect (p=.16) | 12.36 (8.92) | 13.08 (8.78) |  | 16 (9.68) | 11.58 (9.75) | |  |  |
| Sharpe 1997 (one-leg balance, seconds) | 54 | 40 | | 1 year | No sig time x group effect (p=.31) | 3.53 (4.44) | 4.02 (4.47) |  | 4.42 (5.81) | 4.36 (5.24) | |  |  |
| Cwirlej-Sozanska 2018 (Tandem Stance) | 21 | 23 | | 16 weeks | No sig difference between Int and Con (p=.205) | 9.38 (1.53) | 10 (0) |  | 9.82 (0.65) | 9.69 (0.87) | |  |  |
| **Aerobic capacity/endurance n=4 studies** | | | | | | | | | | | | |  |
| Crist 2022 (6MWT) | 210 | 179 | | 24 months | No sig effect (p>.05) | NR | NR | Adjusted regression coefficient: 12.51, 95% CI -1.85, 26.86 | NR | NR | |  |  |
| Kolbe-Alexander 2006 (6MWT, m) | 2 intervention groups (EX1: n = 32 and EX2: n=27) | 22 | | 20 weeks | No sig group x time effect (p>.05) | EX1 = 360.6 (84.4); EX2 = 352.0 (65.7) | EX1 = 410.5 (116.8); EX2 = 474.6 (102.1) |  | 304.8 (122) | 373 (99) | |  |  |
| Owusu 2022 (6MWT, m) | 70 in total - unable to know how many in each arm due to missing data | | | 20 weeks | No sig difference (p=0.1158) | NR | NR | Estimate of change from baseline (int. vs. control): 24.452, SE: 15.469, 95% CI (-6.084, 54.989) | NR | NR | |  |  |
| Moore-Harrison 2008 (VO2 peak) | 12 | 12 | | 16 weeks | Sig increase in Int, vs Con. (P < .05) | 19 | 22.6 |  | 18.4 | 16.7 | |  |  |
| **Gait speed n=3 studies** | | | | | | | | | | | | |  |
| Kolbe-Alexander 2006 (20m, m/s) | 2 intervention groups (EX1: n = 32 and EX2: n=27) | 22 | | 20 weeks | No sig group x time effect (p>.05) | EX1 = 1.15 (0.65); EX2 = 0.87 (0.33) | EX1 = 0.47 (0.48); EX2 = 0.74 (0.35) |  | 1.3 (0.49) | 0.87 (0.6) | |  |  |
| Lipsitz 2019 (gait velocity m/s) | 93 | 87 | | 6 months | No sig difference (p=0.27) | NR | NR | Mean difference (SD): 0.1 (0.2) | NR | NR | | Mean difference (SD): 0.1 (0.2) |  |
| Owusu 2022 (4m walk, sec) | 79 in total - unable to know how many in each arm due to missing data | | | 20 weeks | No sig difference (p=0.3885) | NR | NR | Estimate of change from baseline (int. vs. control): -0.303, SE: 0.350, 95% CI (-0.993, 0.388) | NR | NR | |  |  |
| **Flexibility n=2 studies** | | | | | | | | | | | | |  |
| Wang 2010 (sit and reach test) | 6 | 10 | | 4 weeks | No sig differences at 4 weeks vs baseline in yoga (p=.066) or Con (.681) | NR | NR | Mean difference (SD): -6.06 (5.09) | NR | NR | | Mean difference (SD): -2.72 (17.39) |  |
| Lee 2016 (sit and reach, right) | 2 intervention groups yoga: n=20 and dance: n=20) | 20 | | 36 weeks | No sig time x group effect (p>.05) | yoga = 7.46 (8.56); dance = 4.20 (8.67) | yoga = 14.84 (10.71); dance = 4.40 (2.70) |  | -12 (13.68) | 2.62 (6.65) | |  |  |
| **Multi-component physical function measures n=5 studies** | | | | | | | | | | | | |  |
| Lipsitz 2019 (SPPB total score) | 93 | 87 | | 6 months | No sig difference between Int and Con (p=.62) | NR | NR | Mean difference (SD): 0.05 (0.18) | NR | NR | | Mean difference (SD): 0.18 (0.19) |  |
| Owusu 2022 (SPPB) | 79 in total - unable to know how many in each arm due to missing data | | | 20 weeks | No sig difference (p=0.8174) | NR | NR | Estimate of change from baseline (int. vs. control): 0.075, SE: 0.327, 95% CIs (-0.569, 0.720) | NR | NR | |  |  |
| Stathi 2022 (SPPB) | NR | NR | | 24 weeks | Sig increase in Int vs Con (p=0·014) for overall sample  No sig difference in intervention effect on SPPB by deprivation (p=0.78) | NR | NR | Chi2(4)=1.77 | NR | NR | |  |  |
| Moore-Harrison 2008 (CSPFP, total score) | 12 | 12 | | 16 weeks | Sig increase in Int vs Con (p= .001) | 51.5 (9.8) | 64.3 (11.9) |  | 44.7 (9.3) | 41 (8.1) | |  |  |
| Cwirlej-Sozanska 2018 (SFT - chair stand) | 21 | 23 | | 16 weeks | Sig improvement vs Con group (p=.001) | 11.09 (3.68) | 14.57 (5.63) |  | 12.34 (3.29) | 11.73 (3.09) | |  |  |
| Cwirlej-Sozanska 2018 (SFT - arm curl) | 21 | 23 | | 16 weeks | Sig improvement vs Con group (p=.001) | 11.67 (4.34) | 16.04 (5.13) |  | 13.34 (2.44) | 13.47 (3.31) | |  |  |
| Cwirlej-Sozanska 2018 (SFT - 2 min step) | 21 | 23 | | 16 weeks | Sig improvement vs Con group (p=.001) | 55.23 (15.19) | 91.09 (22.07) |  | 59.95 (15.28) | 60 (23.76) | |  |  |
| Cwirlej-Sozanska 2018 (SFT - chair sit and reach) | 21 | 23 | | 16 weeks | Sig improvement vs Con group (p=.001) | -3.57 (11.43) | 1.71 (10.56 |  | 1.73 (11.72) | 0.21 (14.38) | |  |  |
| Cwirlej-Sozanska 2018 (SFT - back scratch) | 21 | 23 | | 16 weeks | Sig improvement vs Con group (p=.001) | -9.04 (12.18) | -4.04 (10.02) |  | -4.17 (8.39) | -5.69 (8.7) | |  |  |
| Cwirlej-Sozanska 2018 (SFT - 8 foot up and go) | 21 | 23 | | 16 weeks | Sig improvement vs Con group (p=.001) | 9.57 (2.18) | 7 (1.81) |  | 9.04 (2.28 | 10.08 (2.13) | |  |  |
| **Self-reported physical functioning n=5 studies** | | | | | | | | | | | | |  |
| Yin 2021 (basic ADL) | 34 | 15 | | 12 weeks | Sig improvement in FSQ basic ADL in Int vs Con (p=.02) | NR | 11.9 (0.3) |  | NR | 11.2 (1.6) | |  |  |
| Yin 2021 (intermediate ADL) | 34 | 15 | | 12 weeks | No sig effect (p=.47) | NR | 19.9 (5.8) |  | NR | 17.5 (6) | |  |  |
| Kolbe-Alexander 2006 (ADL/IADL) | 2 intervention groups (FS: n = 28 and MS: n=22) | 22 | | 20 weeks | No significant changes were observed for ADL and IADL following intervention | NR | NR |  | NR | NR | |  |  |
| Owusu 2022 (SF-36 Physical Function) | 77 in total - unable to know how many in each arm due to missing data | | | 20 weeks | Sig difference between Int and Con (p=0.0002) | NR | NR | Estimate of change from baseline (int. vs. control): 11.24, SE: 2.94, 95% CI (5.439, 17.040) | NR | NR | |  |  |
| Owusu 2022 (Risk of functional decline - VES-13 score) | 79 in total - unable to know how many in each arm due to missing data | | | 20 weeks | No sig difference between Int and Con (p=0.1776) | NR | NR | Estimate of change from baseline (int. vs. control) :-0.295, SE:0.218, 95% CI (-0.725, 0.135) | NR | NR | |  |  |
| Moore-Harrison 2008 (SF-36 Physical Function) | 12 | 12 | | 16 weeks | Sig improvement in Int vs Con (p=.014) | 81.7 (18.6) | 85.8 (13.6) |  | 69.6 (18.3) | 65 (16.4) | |  |  |
| Sharpe 1997 (perceived change in physical functioning) | NR | NR | | 1 year | Sig increase in Int vs Con (p = .02) | NR | 17.3 |  | NR | 19.1 | |  |  |
| **General physical health n=5 studies** | | | | | | | | | | | | |  |
| Batik 2008 (HbA1c) | 14 | 170 | | ≥ 6 months | No sig difference between Int and Con | 7 (1.5) | NR |  | 7.2 (1.6) | NR | |  |  |
| Vieira 2021 (HbA1c) | 8 | 15 | | 3 months | No sig change in Int (p=0.918) or Con p=0.707) | 7 (1) | 7 (1) |  | 7(1) | 7(1) | |  |  |
| Kolbe-Alexander 2006 (self-reported health status) | 2 intervention groups (FS: n = 28 and MS: n=22) | 22 | | 20 weeks | No sig difference between groups | NR | NR |  | NR | NR | |  |  |
| Kolbe-Alexander 2006 (systolic blood pressure) | 2 intervention groups (FS: n = 28 and MS: n=22) | 22 | | 20 weeks | Sig decrease from baseline to 20 weeks in both EX1 and EX2 vs Con (p=0.013) | EX1 = 148 (13); EX2 = 143.14 (14) | EX1 = 144 (13); EX2 = 137 (15) |  | 147 (13) | 150 (16) | |  |  |
| Kolbe-Alexander 2006 (diastolic blood pressure) | 2 intervention groups (FS: n = 28 and MS: n=22) | 22 | | 20 weeks | No sig effect | EX1 = 90 (10); EX2 = 92 (10) | EX1 = 88 (9); EX2 = 88 (10) |  | 91 (10) | 89 (11) | |  |  |
| Crist 2022 (systolic blood pressure) | 210 | 179 | | 24 weeks | Int blood pressure declined over first 12 months then returned to baseline levels.  Con had greater decrease in systolic and diastolic from baseline to 18 months compared to Int, Con had overall decrease in blood pressure over 24 months | NR | NR | Adjusted regression coefficient: 1.96, 95%CI 3.31;7.22 | NR | NR | |  |  |
| Crist 2022 (diastolic blood pressure) | 210 | 179 | | 24 weeks | Int blood pressure declined over first 12 months then returned to baseline levels.  Con had greater decrease in systolic and diastolic from baseline to 18 months compared to Int, Con had overall decrease in blood pressure over 24 months | NR | NR | Adjusted regression coefficient: 1.73 95% CI -1.04;4.49 | NR | NR | |  |  |
| Kolbe-Alexander 2006 (BMI) | 2 intervention groups (FS: n = 28 and MS: n=22) | 22 | | 20 weeks | Sig decrease in EX1 baseline to 20 weeks, no sig changes in EX2 or control groups | EX1 = 31.7 (7.1); EX2 = 30.31 (6.8) | NR |  | 29 (9) | NR | |  |  |
| Lipsitz 2019 (no. of falls/year) | 93 | 87 | | 6 months | No sig difference between Int and Con (p = .25) | 1.15 (2.2) | 0.46 |  | 0.79 (1.06) | 0.26 | |  |  |
| **Psychological/wellbeing measures n=8 studies** | | | | | | | | | | | | |  |
| **Quality of life n=6 studies** | | | | | | | | | | | | |  |
| Brandao 2021 (WHOQOL-OLD) | 61 | 64 | | 12 weeks | Sig increase in Int (p < .01) but not Con (p=.6) | 85 (10) | 90.4 (9) | Mean difference (SD): 5.4 (9.5) | 84.3 (10) | 83.7 (10) | | Mean difference (SD): 0.6 (8.8) |  |
| Crist 2022 (PQOL) | 210 | 179 | | 24 weeks | Int had sig increase scores at 12, 18, and 24 months from baseline,  compared to Con (p<.001) | NR | NR | Adjusted regression coefficient: 0.52 95%CI 0.25;0.79 | NR | NR | |  |  |
| Lipsitz 2019 (HRQOL - SF12 Physical Component) | 93 | 87 | | 6 months | No sig difference between Int and Con (p=.55) | 40.6 (10.7) |  | Mean difference (SD): 0.22 (0.99) | 40.8 (10.9) |  | | Mean difference (SD): -0.64 (1.04) |  |
| Lipsitz 2019 (HRQOL - SF12 Mental Component) | 93 | 87 | | 6 months | No sig difference between Int and Con (p=.51) | 51.8 (9.8) |  | Mean difference (SD): -1.81 (1.16) | 52.3 (10) |  | | Mean difference (SD): -0.71 (1.19) |  |
| Owusu 2022 (FACT-B) | 78 in total - unable to know how many in each arm due to missing data | | | 20 weeks | Sig difference (p=0.0015) | NR | NR | Estimate of change from baseline (int. vs. control): 6.445, SE: 2.524, 95% CI (1.465, 11.425) | NR | NR | |  |  |
| Stewart 1997 (MOS) | 59 | 30 | | 6 months | Self-esteem improved in the intervention group relative to the  comparison group (F = 4.05, p < .05). No sig differences were found for physical functioning; limitations in  social activities due to health, energy, pain, sleep adequacy;  self-rated health; sense of mastery; psychological well-being; and  life satisfaction. | NR | NR |  | NR | NR | |  |  |
| Yin 2021 (SF-12 Physical Component) | 34 | 15 | | 12 weeks | Sig increase in Int vs Con (p=.04) | 45.6 (9.3) | 48.5 (7.8) |  | 41.9 (11.7) | 44.1 (9.8) | |  |  |
| Yin 2021 (SF-12 Mental Component) | 34 | 15 | | 12 weeks | No sig effect (p=.89) | 52.6 (10.5) | 53.7 (8.8) |  | 56.4 (7.6) | 56.9 (6.9) | |  |  |
| **Depression n=3 studies** | | | | | | | | | | | | |  |
| Crist 2022 (CES-D) | 210 | 179 | | 24 weeks | No sig effect | NR | NR | Adjusted regression coefficient: 0.84 95% CI -0.10; 1.78 | NR | NR | |  |  |
| Lipsitz 2019 (CES-D) | 93 | 87 | | 6 months | No sig difference between Int and Con (p=.69) | 12.3 (8.4) |  | Mean difference (SD): -0.06 (0.69) | 11.5 (8) |  | | Mean difference (SD): -0.47 (0.74) |  |
| Wang 2010 (CES-D) | 7 | 10 | | 4 weeks | No sig difference for yoga (p=.270) or Con (p=.758) | NR | NR | Mean difference (SD): 0.857 (1.86) | NR | NR | | Mean difference (SD): -0.2 (1.99) |  |
| **Other psychological/wellbeing outcomes n=5 studies** | | | | | | | | | | | | |  |
| King 2013 (motivational processes of change) | 20 | 10 | | 4 months | Int sig increased understanding the risks of inactivity (p = .03, effect size = 0.6), committing oneself to being physically active (p = .006, effect size = 0.9), substituting more active alternatives (p = .0004, effect size = 1.2), rewarding oneself for being physically active (p = .03, effect size = 0.7), and reminding oneself to be physically active (p = .009, effect size = 0.9) compared to Con. | NR | NR |  | NR | NR | |  |  |
| Wang 2010 (Morale - Philadelphia Geriatric Center Morale Scale) | 7 | 10 | | 4 weeks | No sig difference for yoga (p=.099) or Con (p=.271) | NR | NR | Mean difference (SD): 2.14 (2.91) | NR | NR | | Mean difference (SD): 0.70 (1.89) |  |
| Wang 2010 (Hope - Herth Hope Index) | 7 | 10 | | 4 weeks | No sig difference for yoga (p=.281) or Con (p=.647) | NR | NR | Mean difference (SD): 1.29 (2.87) | NR | NR | | Mean difference (SD): 0.50 (3.34) |  |
| Wang 2010 (Social Isolation - UCLA Loneliness Scale) | 7 | 10 | | 4 weeks | No sig difference for yoga (p=.289), sig increase for Con (p=0.25) | NR | NR | Mean difference (SD): 0.43 (0.98) | NR | NR | | Mean difference (SD): 0.70 (.82) |  |
| Owusu 2022 (Sleep - Pittsburgh Sleep Quality Index) | 72 in total - unable to know how many in each arm due to missing data | | | 20 weeks | No sig difference (p=0.3986) | NR | NR | Estimate of change from baseline (int. vs. control): -0.507, SE: 0.6, 95% CI (-1.691, 0.676) | NR | NR | |  |  |
| Lipsitz 2019 (balance confidence - Activities-specific Balance Confidence Scale) | 93 | 18 | | 6 months | No sig difference between Int and Con (p=.41) | 69.9 (23.9) | NR | Mean difference (SD): -0.11 (1.77) | 73.6 (22.2) | NR | | Mean difference (SD): -2.02 (1.87) |  |
| Yin 2021 (chronic pain severity - brief pain inventory) | 34 | 15 | | 12 weeks | No sig difference between Int and Con (p=.58) | 2.5 (2.8) | 1.5 (2.1) |  | 2.4 (2.9) | 1.8 (2.4) | |  |  |
| Yin 2021 (chronic pain interference - brief pain inventory) | 34 | 15 | | 12 weeks | No sig difference between Int and Con (p=.07) | 1.8 (2.4) | 0.9 (1.5) |  | 1.9 (2.5) | 1.9 (2.8) | |  |  |

NR = not reported
