# Supplementary material for: The effectiveness and acceptability of physical activity interventions amongst older adults with lower socioeconomic status: a mixed methods systematic review
Source: Int J Behav Nutr Phys Act. 2024 Oct 22;21:121. doi: 10.1186/s12966-024-01666-8 (PMC11495005; doi:10.1186/s12966-024-01666-8)
Supplement: Supplementary file 8 — Additional file 8: Descriptive themes, sub-themes and supporting findings and quotes identified in the thematic synthesis [file 12966_2024_1666_MOESM8_ESM.docx]

**Additional file 8. Descriptive themes, sub-themes and supporting findings and quotes identified in the thematic synthesis**

| **Descriptive Theme** | **Descriptive Sub-themes** | **Findings and supporting quotes** |
| --- | --- | --- |
| Perceived benefits of engaging in physical activity | Physical benefits | Participants described how engaging in physical activity interventions had led to several physical health benefits including improvements in strength, mobility, flexibility and balance, weight loss, increased energy, and helping with existing health issues.  *“Yes I feel more mobile on my feet” (1)*  *“I can go a bit further down to touching my toes, I’m not quite there yet but we’re getting there” (1)*  *“I find my arm and shoulder are getting more flexible” (1)*  *“I’ve found my balance has increased tremendously … my legs are stronger and are more able to [adapt to] uneven sidewalks” (2)*  *“We’re doing balancing exercises that are helping us a lot, my balance seems to be getting better.” (3)*  *“I can't bend over too much; I have knee issues, but if you can do that for 1 hour and 30 minutes out there every day. It just seemed to take everything away.” (4)*  *“Yeah, I lose a lot. Yeah, it makes me lose a lotta weight. I don’t eat as much as I used to eat so, yeah, everything is fine.” (3)*  *“Once you start doing all the movements and stuff like that, it’s like you wake up, your body feels generated to go on, and then you can do something else outside.” (2)*  Participants also reported having *“quicker recovery after being sick” or “after surgery” (5)* |
|  | Psychological benefits | Participants reported numerous psychological benefits such as improvements in mood, increased confidence, feeling more relaxed and having better sleep.  *“When they ask me how it is, I say, “I feel great” or “I feel terrific.” (2)*  *“Feeling quite alright after, tired but feeling a bit refreshed as well at the same time good, it uplifts your mood”. (1)*  *“It teaches you, you know, just relax and go with the flow and, you know, let it trickle down. And, you know, and I just enjoy it’s really— It’s very helpful, it really is, it makes you feel so much better right. I’m so happy that we got it here.” (2)*  *“There is for me a definite feeling of well-being that I didn’t have before…. My body parts are holding together without any strain. And I think that is important for older people, it gives you confidence in moving.” (2)*  *“I think you are calmer and you try to react to the people like if some people really irk you but you don’t get as pissed off as we say. No it sort of like calms you down …” (2)*  *“I noticed when since I’ve been doing the Tai Chi I used to yell at my husband all the time … and now I just won’t say anything, I’ll just sort of pass … at home it’s not as stressful. So it’s a lot better.” (2)*  *“You don't think about what's on your mind. It's your own little world.” (4)* |
|  | Social benefits | Participants reported that they had met new people and made new friends and felt a sense of social connectedness. This also sometimes extended beyond the intervention as well. Perceived social benefits were also often a motivator to engage in the intervention in the first place.  *“I think it’s just getting together as a group and you know socializing and doing these exercises and having fun doing it together." (3)*  *“people looking out for you and asking where you were” (6)*  *“Group serves as motivator for exercising” (5)*  *“And the thing is, if somebody’s sick, you wanna see if they’re okay and if you can do something for them. Because it’s like we try to take care of one another. And I think that’s really... We’ve just formed a bond with all of us and it’s wonderful.” (2)*  *“group activity can be a great draw especially if alone” (6)*  A participant had reported that they received *“support from [new] friends after surgery”* and some participants had started to also plan other social activities outside of the intervention such as museum trips (5). |
|  | Wider benefits | Some participants talked about other benefits beyond the intervention such as engaging in walking as active transport, feeling more confident to do other activities outside of the intervention as well as increased knowledge of opportunities to be active within their local area.    *“I feel like I’ve got a little bit more confident, cos I’ve started going to … over there and one time I couldn’t get across the road for traffic, erm now I’ve got to a point where I can get across and see everything coming and get across the road and go to…” (1)*  Some participants reported that they now saw walking as way of socialising with others or a mode of transport (Rodriguez Espinosa) and that participants had also started to prefer to walk to some places rather than take other forms of transport. (7)  Participants also reported an increased awareness of opportunities to being physically active within their communities such as parks and walking trails. (5) |
| Perceptions of physical activity in older age | Age-appropriate activity | Participants reported perceptions of gentler exercise as being more suited to older adults and this was often a draw for them to engage with the intervention initially.  *“It was a great way to stretch your muscles without being harsh about it.” (2)*  *“good for seniors health condition because the actions/practices are slow” (2)*  *“…thought because it is dance for older people it would be a more gentler exercise rather than going to the gym” (1)* |
|  | It’s never too late | Participants reported that benefits could still be gained from doing physical activity in their later years and that they still had the power to change things for themselves.  *“Elderly people tend to be kind of pushed aside we’re all, we’re not 19, we don’t have these fabulous bodies and they kind of look at us like we can’t do anything, but this has really shown us.” (2)*  *“I think it gives me, let’s put it that way, more of a positive outlook that something can be done. Rather than just saying, “Oh, gosh I’m getting old and this is falling off, and that’s falling down. This is wrinkling up.” It’s an idea that well, yeah this is happening, but I can do something about it. There’s hope. There’s something there that can be done to counteract that.” (2)*  *“Because I knew that I was in danger of getting erm more or less seat bound so I needed some activity” (1)*  *“I think as the years pile on, balance and coordination become much more difficult, so anything that would help readdress the balance a bit, or anything that would help would be very useful” (1)* |
| Importance of setting | Convenience | Participants described the importance of how convenient the location of any physical activity sessions were to get to. Having a location that was easy to get to was a facilitator towards engaging in the physical activity intervention, whilst longer travel distances were barriers to participation.  *“because all the ones that were there, they were used to going to the bingo anyway, we can all get there ... Because it’s central.” (8)*  *“They know the hall. For me, I knew I’d get a bus no problem, ken [know] what I mean.” (8)*  *“Because it was in the bingo hall, it is just so convenient.” (8)*  *“I don’t have a car so it would be very difficult for me to get to, you know, some place like that. So to me it’s like a marvelous thing and I, you know what more could you ask for.” (2)*  One study reported barriers faced by those living in a big city many of which centred around having to get public transport, rush hour and needing to have the physical capacity to use this. (6) |
|  | Familiarity | Participants described how the familiarity of the setting, not just the location itself but also being amongst their peers helped them to feel more comfortable and was a facilitator to engaging in the intervention.  *“Well, it’s a bingo hall. I know everybody in it. It wasn’t going to cause me any embarrassment because everybody knew me and I knew everybody who was coming too, ken [know] what I mean?” (8)*  *“There were women there that definitely wouldn’t have the confidence to go along to a regular class and walk in on their own and go to a regular class. I think being in a familiar environment, the bingo environment, really helped them to have the confidence to actually turn up in the first place.” (8)*  *“I just, I feel out of place in a gym. You know. And older women, they just feel, as young, fit people looking at you ... Old women at the gym just dinnae [don’t] fit ...” (8)*  One study found that a key barrier to engaging in physical activity interventions was not being amongst people of similar age or race, as they would not have a shared understanding of difficulties in trying to be physical active. (9) |
|  | Cost | Participants described how cost was often a barrier to participating in physical activity interventions, in terms of the cost of taking part in the exercise classes themselves, and any transports costs of getting to these.  *“I was looking into different program … and of course, my main problem with all of it was the cost.” (2)*  Providing physical activity interventions free of charge to participants was a facilitator to engagement (2, 7)  One study reported that participants having to get public transport including the cost of this was a barrier to participating (6)  One study also found participants perceived cost-saving as a benefit of engaging in a gardening intervention:  *“You have a lot of problems. Your paying bills here and there and you're stretching every penny. Yes that's the reason why you do gardening and anything else you could do, it will help you but also you can save a few dollars if you can get your plants free.” (4)* |
| Time is an issue | Competing commitments | Participants reported that the timings of physical activity classes were important due to competing demands to their schedule, such as caring for others and attending medical appointments.  Barriers to participation were “time conflicts with medical appointments” (2) which was particularly an issue for those with multiple chronic conditions (6). Participation in intervention also “interfered with their ability to care for their parent” (2) as well as “roles of day care and baby-sitting” for their grandchildren (6)  *“not as good in morning since grandkids sleeping” (6)* |
|  | Need for efficiency | Combining physical activity with other activities was seen as a solution to overcoming barriers of scheduling conflicts and competing commitments, helping to make better use of participants’ time.  *“Because our [children’s] program starts at 1 . . . So we think 1:30 [for Tai Chi] when we settle the kids, then we can do it.” also, “if you want to leave them [children] alone, you have to have the kids program on” (6)*  *“Monday mornings good because right after is another program, bingo” and “we can also have another class Friday right after another function” also, “[we] are already here so good time for another [TC] class” (6)*  *“afternoons good, can come with wife” (6)*  Some participants also expressed how classes were “too short” and wanted them “to be longer” (2, 10) |
| Instructors play a big role | Likeability | Some participants reported that it was important for them to “like the instructor” (6) and that having instructors who were well liked was seen as a real strength of the intervention by participants (7). Participants also expressed the importance of instructors helping them to feel comfortable.  *“[Instructors] have been so welcoming as well to everybody and made people feel at ease and I think that’s been a big help”. (1)* |
|  | Relatability | Participants described how it was important for instructors leading the interventions to have some common ground and shared understanding of their experiences, particularly with regards to age. Peer leaders were seen as an effective way of providing this, particularly beyond the timeframe of the intervention itself, to increase sustainability.  *“instructors need to relate and vice versa” and “sometimes young [age of instructor] is a barrier” (6)*  Participants spoke of how they wanted someone who know *“what it feels like to be in an aging body”* and talked of a past instructor who *“was injured and older, in pain, and participants knew that the instructor could relate to them” (6)*  One study (7) found success when instructors had handed the running of classes over to peer leaders, with increased frequency of exercises classes, and one of the peer leaders then going on to lead classes at another community centre that had not originally been involved with the initial intervention. |

References

1. Britten L, Pina I, Nykjaer C, Astill S. Dance on: a mixed-method study into the feasibility and effectiveness of a dance programme to increase physical activity levels and wellbeing in adults and older adults. BMC Geriatrics. 2023;23(1):48.

2. Lo OY, Conboy LA, Rukhadze A, Georgetti C, Gagnon MM, Manor B, et al. In the Eyes of Those Who Were Randomized: Perceptions of Disadvantaged Older Adults in a Tai Chi Trial. Gerontologist. 2020;60(4):672-82.

3. VanRavenstein K, Davis BH. When more than exercise is needed to increase chances of aging in place: qualitative analysis of a telehealth physical activity program to improve mobility in low-income older adults. JMIR aging. 2018;1(2):e11955.

4. Wang D, Glicksman A. “Being Grounded”: Benefits of Gardening for Older Adults in Low-Income Housing. Journal of Housing For the Elderly. 2013;27(1-2):89-104.

5. Rodriguez Espinosa P, King AC, Blanco-Velazquez I, Banchoff AW, Campero MI, Chen WT, et al. Engaging diverse midlife and older adults in a multilevel participatory physical activity intervention: evaluating impacts using Ripple Effects Mapping. Transl Behav Med. 2023;13(9):666-74.

6. Manson JD, Tamim H, Baker J. Barriers and Promoters for Enrollment to a Community-Based Tai Chi Program for Older, Low-Income, and Ethnically Diverse Adults. J Appl Gerontol. 2017;36(5):592-609.

7. Sharpe PA, Jackson KL, White C, Vaca VL, Hickey T, Gu J, et al. Effects of a one-year physical activity intervention for older adults at congregate nutrition sites. The Gerontologist. 1997;37(2):208-15.

8. Evans JM, Connelly J, Jepson R, Gray C, Shepherd A, Mackison D. A physical activity intervention in a Bingo club: Significance of the setting. Health Education Journal. 2018;77(3):377-84.

9. Hammerback K, Felias-Christensen G, Phelan EA. Evaluation of a telephone-based physical activity promotion program for disadvantaged older adults. Prev Chronic Dis. 2012;9:E62.

10. Stewart AL, Gillis D, Grossman M, Castrillo M, Pruitt L, McLellan B, et al. Diffusing a research-based physical activity promotion program for seniors into diverse communities: CHAMPS III. Prev Chronic Dis. 2006;3(2):A51.
