# Supplementary material for: The effectiveness and acceptability of physical activity interventions amongst older adults with lower socioeconomic status: a mixed methods systematic review
Source: Int J Behav Nutr Phys Act. 2024 Oct 22;21:121. doi: 10.1186/s12966-024-01666-8 (PMC11495005; doi:10.1186/s12966-024-01666-8)
Supplement: Supplementary file 9 — Additional file 9: Implications for intervention development and findings from which they were derived [file 12966_2024_1666_MOESM9_ESM.docx]

**Additional file 9. Implications for intervention development and findings from which they were derived**

| **Implications for intervention development** | **Findings from acceptability synthesis** | **Findings from effectiveness synthesis** |
| --- | --- | --- |
| ***Interventions should consider how to foster social connectivity.***  Physical activity interventions could provide opportunities for participants to mix with others, particularly with those they share experiences with. | - Anticipated social benefits were often a motivator for individuals engaging with physical activity interventions in the first instance. - Being amongst peers helped participants to feel at ease and was a facilitator to engaging in physical activity interventions. | - Positive effects on physical activity levels for interventions delivered in groups (1-3) - Positive effects on physical function outcomes for interventions utilising social support as a BCT (4, 5) |
| ***Interventions should consider how to emphasise the benefits of physical activity.***  Future interventions could emphasise the different benefits that can be gained from engaging in physical activity to improve both intervention acceptability and effectiveness. | - Participants reported many different perceived benefits from engaging in physical activity interventions, including physical, psychological and social benefits. | - Interventions which emphasised the health benefits of physical activity were effective at increasing physical activity levels (6, 7). |
| ***Physical activity could be combined with other activities to make more efficient use of participants’ time.***  Future development of physical activity interventions could carefully plan the scheduling of physical activity programs.  For example, combining physical activity with other activities that lower SES older adults are already doing (e.g. by holding them in locations where individuals already go to do other things), or target increasing physical activity for other purposes (e.g. transportation). | - Competing commitments were often reported as barriers to engaging in physical activity interventions. - Increased levels of walking as active transport were often seen as a wider benefit of engaging in physical activity interventions. | - Interventions were found to have positive effects on walking for transportation purposes but not leisure (2, 3). |
| ***Interventions should consider using locations that are accessible and familiar.***  Future physical activity interventions could be held in settings that are easy to access to reduce the barriers of longer travel distances, and settings that lower SES older adults are already accustomed to rather than less familiar settings like gyms. | - Location was an important factor when it came to intervention acceptability, both in terms of convenience and familiarity with the setting. | - Most studies were conducted within community settings, although it was difficult to determine how familiar or convenient settings were to get to for participants. - Positive effects on physical activity and physical function for some interventions taking place in participants own living facilities (2, 8, 9), but not others (10, 11) - One study mentioned that the setting was a 30-minute drive from participants’ housing, this intervention had no effect on physical activity levels (12) |
| ***Interventions should consider how to minimise costs to participants.***  Future interventions could minimise costs to those taking part, for example through providing sessions free of charge and also reducing travel costs. | - Cost was often reported as a barrier to participating in physical activity interventions in terms of the cost of taking part in the exercise classes themselves, and any transports costs of getting to these. - Providing programs free of charge was a facilitator to engagement. | - Positive effects on mobility outcomes for an intervention which provided free transport for some participants (13). - Positive effect on strength and self-report physical function of strength and balance intervention provided free of charge (14). |
| ***Interventions should consider using leaders who have shared characteristics with participants.***  Future interventions could have peer leaders delivering them. | - Having instructors who shared similar experiences and who participants could relate to, was a key facilitator to engaging in physical activity interventions. | - Peer-led physical activity interventions were effective at increasing physical activity levels (1, 3), QoL (1), upper body strength and positive perceptions in change in physical functioning (14). |

References

1. Crist K, Full KM, Linke S, Tuz-Zahra F, Bolling K, Lewars B, et al. Health effects and cost-effectiveness of a multilevel physical activity intervention in low-income older adults; results from the PEP4PA cluster randomized controlled trial. International Journal of Behavioral Nutrition and Physical Activity. 2022;19(1):75.

2. Patch CM, Conway TL, Kerr J, Arredondo EM, Levy S, Spoon C, et al. Engaging older adults as advocates for age-friendly, walkable communities: The Senior Change Makers Pilot Study. Translational Behavioral Medicine. 2021;11(9):1751-63.

3. Prins RG, Kamphuis CBM, Van Lenthe FJ. The effects of small-scale physical and social environmental interventions on walking behaviour among Dutch older adults living in deprived neighbourhoods: results from the quasi-experimental NEW.ROADS study. International Journal of Behavioral Nutrition and Physical Activity. 2019;16(1):133.

4. Yin Z, Martinez CE, Li S, Martinez M, Peng K, Land WM, et al. Adapting Chinese Qigong Mind-Body Exercise for Healthy Aging in Older Community-Dwelling Low-income Latino Adults: Pilot Feasibility Study. JMIR Aging. 2021;4(4):e29188.

5. Stathi A, Greaves CJ, Thompson JL, Withall J, Ladlow P, Taylor G, et al. Effect of a physical activity and behaviour maintenance programme on functional mobility decline in older adults: the REACT (Retirement in Action) randomised controlled trial. The Lancet Public Health. 2022;7(4):e316-e26.

6. King AC, Bickmore TW, Campero MI, Pruitt LA, Yin JL. Employing virtual advisors in preventive care for underserved communities: results from the COMPASS study. J Health Commun. 2013;18(12):1449-64.

7. Stewart AL, Mills KM, Sepsis PG, King AC, McLellan BY, Roitz K, et al. Evaluation of CHAMPS, a physical activity promotion program for older adults. Ann Behav Med. 1997;19(4):353-61.

8. Brandão GS, Brandão GS, Sampaio AAC, Damas Andrade L, Fonseca AL, Campos FKR, et al. Home physical exercise improves functional mobility and quality of life in the elderly: A CONSORT-prospective, randomised controlled clinical trial. Int J Clin Pract. 2021;75(8):e14347.

9. Moore-Harrison TL, Speer EM, Johnson FT, Cress ME. The effects of aerobic training and nutrition education on functional performance in low socioeconomic older adults. J Geriatr Phys Ther. 2008;31(1):18-23.

10. Lipsitz LA, Macklin EA, Travison TG, Manor B, Gagnon P, Tsai T, et al. A Cluster Randomized Trial of Tai Chi vs Health Education in Subsidized Housing: The MI-WiSH Study. J Am Geriatr Soc. 2019;67(9):1812-9.

11. Wang DS. Feasibility of a Yoga Intervention for Enhancing the Mental Well-Being and Physical Functioning of Older Adults Living in the Community. Activities, Adaptation & Aging. 2010;34(2):85-97.

12. Owusu C, Margevicius S, Nock NL, Austin K, Bennet E, Cerne S, et al. A randomized controlled trial of the effect of supervised exercise on functional outcomes in older African American and non-Hispanic White breast cancer survivors: Are there racial differences in the effects of exercise on functional outcomes? Cancer. 2022;128(12):2320-38.

13. Bann D, Chen H, Bonell C, Glynn NW, Fielding RA, Manini T, et al. Socioeconomic differences in the benefits of structured physical activity compared with health education on the prevention of major mobility disability in older adults: the LIFE study. J Epidemiol Community Health. 2016;70(9):930-3.

14. Sharpe PA, Jackson KL, White C, Vaca VL, Hickey T, Gu J, et al. Effects of a one-year physical activity intervention for older adults at congregate nutrition sites. The Gerontologist. 1997;37(2):208-15.
